# Workplace Strategies to Reduce Burnout in Veterinary Nurses and Technicians: A Delphi Study

**DOI:** 10.3390/ani15091257

**Published:** 2025-04-29

**Authors:** Angela J. Chapman, Pauleen C. Bennett, Vanessa I. Rohlf

**Affiliations:** School of Psychology and Public Health, La Trobe University, Bundoora, VIC 3086, Australia; pauleen.bennett@latrobe.edu.au (P.C.B.); v.rohlf@latrobe.edu.au (V.I.R.)

**Keywords:** workplace mental health, animal care professional, leadership, wellbeing, retention, workload, regulation, utilisation

## Abstract

Veterinary nurses and technicians are at risk of burnout, which negatively impacts mental and physical health, turnover, and patient care. Workplace contributors to burnout have been identified in this population, but little is known about best practice management strategies. This study used the Delphi method to explore barriers to addressing burnout and develop expert recommendations for workplace management strategies. Forty participants with a minimum of 5 years’ industry experience in leadership, or wellbeing, were recruited via purposive sampling from the USA, UK, Australia, New Zealand, and Canada. Participants completed two anonymous, online, mixed-methods surveys between October 2024 and January 2025. Qualitative survey data were analysed using content analysis to identify codes and categorise solutions. Quantitative data were analysed using descriptive statistics. Barriers to addressing burnout included industry-wide barriers, such as lack of, or unclear, regulation and lack of leadership knowledge, and clinic-specific barriers, such as poor team culture, unwillingness for change, and existing burnout. Thirty-nine solutions were developed and rated as being highly, or very highly effective. These focused on themes such as improving communication, developing progression pathways, and providing leadership training and support. Existing workplace barriers must be evaluated prior to selecting strategies, to maximise effectiveness in specific contexts.

## Simple Summary

Veterinary nurses and technicians experience burnout, which affects their mental and physical health, the likelihood of leaving their job, and their quality of work. Burnout is caused by workplace issues, and existing research has identified specific causes in veterinary nurses and technicians. But little is known about how to address these issues within veterinary clinics in ways that are both effective and feasible. To address this gap, we consulted a panel of 40 veterinary nurse and technician leadership or wellbeing experts to seek their opinions on the challenges to addressing burnout and recommended management approaches. Responses were collected via two anonymous surveys between October 2024 and January 2025. The lack of, or unclear, regulation, poor leadership knowledge, poor team culture, an unwillingness to change, and existing burnout were found to make burnout management more difficult. Thirty-nine possible solutions, with recommended actions, were developed. These focused on improving communication, progression pathways, delegation, and leadership. Panel experts rated the proposed solutions as being highly, or very highly effective. When making changes in the workplace, current workplace problems, such as poor team culture and poor leadership, need to be considered first, as these will reduce the likelihood of success.

## 1. Introduction

Veterinary team members are prone to burnout, with veterinary nurses and technicians (VN/Ts) at particular risk [1]. The existing body of evidence on burnout in veterinarians and the unique contributors to burnout in their role is growing [2,3], however, research specific to burnout in VN/Ts is lacking [4]. A recent study found that VN/Ts were twice as likely to experience high/very high levels of burnout compared to the general population, with exhaustion being a key contributor [5]. The same study identified that having a good work-life balance was the biggest predictor of low burnout levels [5]. Burnout has well documented negative physical and mental health consequences for the individual, with potential welfare implications for veterinary patients under their care. Burnout is also a known contributor to intentions to leave, thus leading to increased staff turnover and increased economic costs for veterinary practice owners [6,7,8]. Effective prevention and management of burnout within VN/Ts is, therefore, critical to ensuring team wellbeing, business sustainability, and patient outcomes.

Burnout is defined as a syndrome resulting from chronic workplace stress which has not been adequately managed [9] and is, therefore, considered an occupational health issue. According to the International Labour Organisation (ILO), a United Nations agency that sets international labour guidelines for its 187 member states, a safe and healthy work environment is a fundamental right for employees [10]. Furthermore, the World Health Organisation (WHO) guidelines on mental health at work, written in consultation with the ILO, state that employers have a responsibility to ensure that psychosocial risks, which can contribute to burnout, are managed within the workplace to protect employee’s mental health [11]. Psychosocial risks are defined as “anything in the design or management of work that increases the risk of work-related stress” [12] (psychosocial risks section). This may include a lack of work variety, lack of job control or inflexible hours, poor communication, lack of support, and poor work-life balance [12]. A shared responsibility exists between the workplace and the employee to manage and protect the mental health and wellbeing of individuals through preventing burnout.

Burnout contributors can be divided into risk and protective factors, with protective factors describing aspects of the workplace that can help to reduce an individual’s risk of experiencing burnout. Protective factors in VN/Ts have been found to include manageable workloads, teamwork and support, clear and respectful communication, appropriate staffing levels, recognition of work, and knowledge of making a difference to clients and patients [13]. Risk factors describe aspects of the workplace that increase an individual’s risk of experiencing burnout. These factors may be experienced across a range of industries, for example, co-worker incivility [14], or may be unique to individuals working in animal care roles, for example, exposure to animal abuse and euthanasia [15]. In VN/Ts, risk factors have been found to include high workloads, lack of skill utilisation, negative team culture, unclear communication, poor management or leadership, lack of schedule control, poor remuneration, lack of progression opportunities, negative client interactions, and lack of appreciation [13,16]. Some of these contributors, including high job demands, low job control, lack of support, and team conflict in VN/Ts, were identified in studies published over 10 years ago and, therefore, awareness of these problems is not new [17,18]. Despite this, recent research suggests that they are still being inadequately managed [13,16]. In addition to understanding the contributors to burnout it is, therefore, necessary to identify any barriers and enablers that may prevent, or support, organisations in addressing these contributors, so as to maximise the chances of success.

Barriers are factors that impede the ability of people leaders to address burnout risk factors. These are multifactorial and may be internal to the workplace. This includes barriers related to the interventions themselves, for example, technology that is not suited for the intended use; barriers related to the process of implementation, for example a lack of planning; or barriers related to the characteristics of the team or workplace, for example opposing values within the team. Barriers may also be external to the workplace, related to industry or legislative limitations, for example a lack of standardised educational competencies for professional licensing requirements [19]. Enablers are factors that facilitate people leaders to manage burnout. These may also arise from internal workplace sources, for example clear goals that have been collaboratively developed; or external sources, for example existing research to help understand the impact of changes [19].

Burnout prevention strategies can be classified as primary and secondary interventions, which may be organisational or individual focused, and tertiary interventions, which are generally individual focused [20]. Primary interventions are aimed at addressing the antecedents of burnout and preventing the onset of signs. Secondary interventions focus on identifying signs of burnout in the early stages and preventing the development of longer-term impacts. Tertiary interventions are aimed at addressing existing burnout and minimising the long-term effects through healing and rehabilitation [20]. Implementation of primary and secondary interventions by the organisation requires an understanding of the unique contributors to burnout in VN/Ts.

Existing research exploring burnout in VN/Ts includes recommendations on strategies that can be implemented within the workplace to better support individuals. These include providing opportunities for professional development, greater control over shift pattern and length, and management training on development of positive interpersonal relationships [17,21,22]. No studies to date, however, have explored barriers to implementation. Furthermore, existing studies typically include researcher recommendations to address burnout [21,22,23,24]. While these recommendations are informed by research findings derived from VN/Ts, they have not been evaluated for their perceived effectiveness or feasibility. Gaining this information, particularly from experienced wellbeing and leadership experts in the veterinary industry, is critical to ensure best practice recommendations target the known risk and protective factors and are also evaluated as effective and feasible.

Delphi studies are a research method commonly used in the development of human healthcare recommendations, allowing researchers to gain consensus through expert opinion [25]. The Delphi technique has been used in studies to develop workplace guidelines for the prevention of mental health problems [26], as well as to rate the importance of factors in the occurrence of burnout in nurses [27], and is therefore well-suited to research in the area of VN/T burnout. The Delphi method is used to collect objective opinions from participants in geographically diverse locations, across successive iterative rounds, to develop recommendations based on collective expert views. Delphi studies are particularly useful in areas where limited research exists and consensus is absent, and the technique transforms expert opinions into group consensus to elevate the quality of research evidence [25]. The lack of existing research on barriers, enablers, and feasibility of management strategies to address burnout in VN/Ts makes this an ideal framework for developing recommendations in this area.

The aim of this study was to employ a Delphi method to develop effective and feasible organisational management strategies to address known contributors to burnout in VN/Ts. This addressed the existing literature gap by producing recommendations based on expert consensus. This was achieved by, first, identifying perceived barriers and enablers to addressing the contributors, then, second, by exploring perceived ease of implementation and effectiveness of proposed strategies in addressing burnout in the workplace.

## 2. Materials and Methods

The Delphi method [28] was used to conduct the study, and it is reported following the Delphi studies in social and health sciences—recommendations for an interdisciplinary standardized reporting (DELPHISTAR) guideline [29].

### 2.1. Research Design

**Figure 1:**
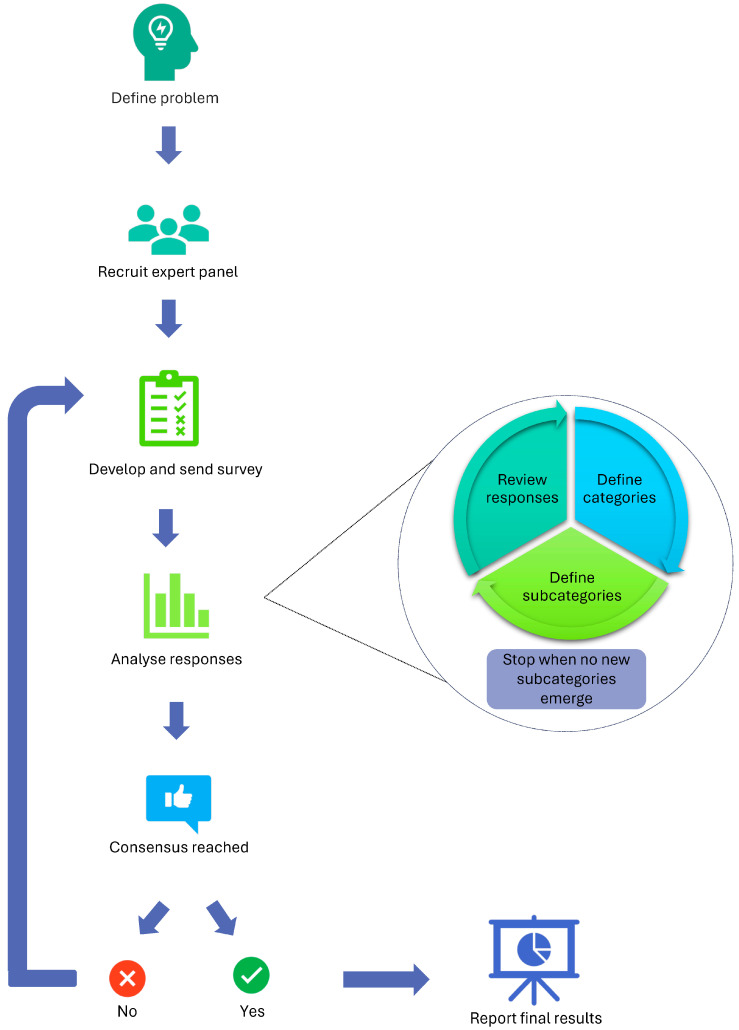
Delphi technique flow chart.

The Delphi method is a structured consultation process whereby a panel of experts contribute their views on an issue via an anonymous survey. Surveys incorporate both quantitative and qualitative questions to allow experts to rate items and provide feedback on their reasoning [25]. Panel members then revise their views through multiple survey rounds based on summarised feedback and responses from other participants, until consensus is reached. Anonymity is maintained throughout the process to minimise risk of bias resulting from perceptions of dominance and group conformity [25]. The current study comprised two rounds of data collection, with additional planned rounds being cancelled due to consensus being reached. In this study, consensus was defined as at least 75% endorsement by the panel across two consecutive ratings, following guidelines from similar research [27,30]. This process can be seen in Figure 1.

### 2.2. Participant Recruitment

Purposive sampling was utilised to identify experts with a minimum of five years’ experience in VN/T leadership, leadership consulting, or wellbeing, as utilised in similar studies [25,31]. Potential participants were identified through their involvement in, or contributions to, VN/T wellness publications, conference presentations, veterinary industry wellbeing initiatives and awards, VN/T wellbeing podcasts, LinkedIn profiles, and existing professional networks of the researchers. Potential participants were contacted via email or LinkedIn and invited to contribute to the study. The panel size in Delphi studies is recommended to be greater than 20, after taking into account attrition across survey rounds [30]. Out of 84 experts invited, 40 consented to participate in the initial round. Participant recruitment was not directed toward specific countries, however, all recruited participants belonged to nations considered as the global West and English speaking. Participants who completed each round were invited to participate in subsequent rounds.

### 2.3. Measures

The aim of Round 1 was to explore barriers, enablers, and proposed strategies to address risk and protective factors for burnout in VN/Ts. The survey comprised a mixture of four questions which were developed by the authors. Participants were asked to respond to the same four questions for each of 10 risk and three protective factors derived from the findings of Chapman et al. [13,16]. For the 10 risk factors, Question 1 asked participants to rate how easy, or difficult, they believe it is to address the issue. Responses were rated on a 5-point Likert scale from ‘very difficult’ to ‘very easy’. Three open-ended questions then asked participants to identify (1) what, if any, barriers they are aware of that increase the difficulty for managers or leaders to address the issue, (2) what, if any, enablers they are aware of that support managers or leaders to address the issue, and (3) what solutions or strategies they are aware of that effectively address the issue. For the three protective factors, the same questions were asked but rephrased to reflect the protective aspect. For example, the words “to address this issue” were replaced with “to promote this factor”. The final open-ended question in the protective factors section removed the word “solutions” and sought strategies to promote the protective factor only, as solutions for protective factors are not required (Supplementary Materials S1). Responses to the questions in Round 1 were analysed and informed the development of solutions to the 10 risk factors, and promotions strategies for the three protective factors, which were then reviewed in the second round of data collection.

The aim of Round 2 was to rate the ease with which each solution or promotion strategy, developed in Round 1, could be implemented, as well as to rate the perceived effectiveness of the solutions in addressing the 10 risk factors. The survey comprised a mixture of three questions developed by the authors. Participants were asked to respond to the same three questions for each of the 10 risk factors, and two of those three questions for the three protective factors. Rating questions asked participants to rate how easy, or difficult, they believed it would be to implement each solution, for the risk factors, and each strategy, for the protective factors, as well as how effective, or ineffective, they believed the solutions for each risk factor would be. Items were measured on 5-point Likert scales ranging from ‘very difficult’ to ‘very easy’ for ease of implementation, and from ‘very ineffective’ to ’very effective’ for effectiveness of solutions. Open-ended questions asked participants to provide feedback on proposed improvements to both solutions and strategies (Supplementary Materials S2).

### 2.4. Procedure

The study was conducted in accordance with the National Statement on Ethical Conduct in Human Research, and ethical approval was obtained from the Human Ethics Low Risk Committee at La Trobe University, Australia (approval number HEC24265). Data for Rounds 1 and 2 were collected between 7 October 2024 and 13 January 2025.

A link to each Round of the survey was emailed to participants and responses were collected using QuestionPro version 2 (Austin, TX, USA) [32]. Prior to receiving the first survey link, participants were required to sign a participation consent form which included advice to contact their local healthcare provider if they experienced any lasting negative emotions after taking part in the study. Links to international helplines were also provided. Each survey was initially open for two weeks, with a short time for data analysis scheduled between rounds to maintain participant engagement and reduce attrition. Closure of both survey rounds was extended by 10 days (Round 1) and one month (Round 2) in order to allow for increased participation, and account for the busy December holiday period (Round 2). Email reminders were sent to participants that had not yet completed the survey two days before the initial survey closure date, and then again after the initial closure date to inform remaining participants of the extended deadline.

A summary of responses was circulated to participants following data analysis of each round to allow participants to compare their responses with those of the collective panel. The Round 1 summary document outlined the key barriers and enablers that had been identified from participant responses, as well as the mean ease-of-management rating for each risk and protective factor. After Round 2, the summary document provided an overview of the ease and effectiveness ratings for solutions proposed in Round 1, as well as highlighting notable findings from the data.

### 2.5. Data Analysis

Data from open ended questions in Round 1 were analysed using NVivo version 15 (Denver, CO, USA) [33] and coded using conventional content analysis [34] to identify and describe the emerging categories. This approach is recommended when existing research on the area is limited, as it identifies patterns and recurrent viewpoints among participant responses to open-ended questions, without exposing participants to preconceived theories or ideas [34]. An iterative approach was taken where one author (AC) read the responses in full and then reviewed them multiple times to identify and refine categories and sub-categories until no novel phenomena emerged. In contrast to thematic analysis which relies on interpretive analytical approach by the researcher to contextualise the data, content analysis takes a descriptive analytical approach, coding and categorising data to identify trends and patterns of words [35]. Risk of bias resulting from the epistemological perspectives of the researcher is, therefore, much lower. Debriefs were conducted between the primary author and the last author (VR) to ensure that context and clarity of the data were accurately represented in the coding, and to further reduce the risk of bias.

Data from open ended questions in Round 2 were analysed using directed content analysis [34] to validate and extend the existing findings from Round 1. Directed content analysis utilises existing themes, which were developed in Round 1, to provide a framework with which to design and analyse questions based on the existing categories [34]. The same iterative process was followed to categorise data into the existing codes developed in Round 1, with debriefs to increase validity of the process [34].

Quantitative data from Rounds 1 and 2 were analysed using descriptive statistics, with means and standard deviations reported for each item. Missing data were managed by calculating the central tendency based on the number of participant responses to each item and excluding missing responses. Percentages and frequency counts for each Likert scale rating were also calculated and used to determine whether consensus had been reached.

## 3. Results

### 3.1. Participants

**Figure 2:**
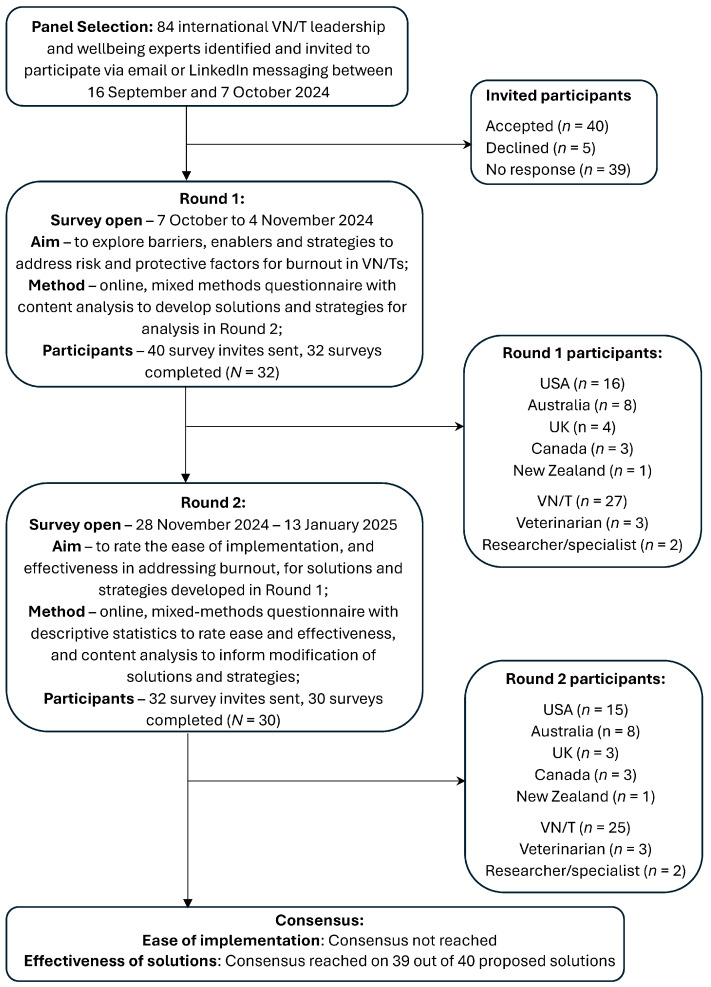
Flow diagram of Delphi study process and participants including country of residence and professional background.

Forty experts were recruited from the United States of America (USA) (*n* = 18), Australia (*n* = 9), United Kingdom (UK) (*n* = 7), Canada (*n* = 4), and New Zealand (*n* = 2). The majority of participants were VN/Ts (*n* = 33), with the remainder either veterinarians (*n* = 3), or veterinary wellbeing experts without a veterinary clinical qualification (*n* = 3). A total of 32 and 30 participants completed the survey in Rounds 1 and 2 respectively (Figure 2).

### 3.2. Round 1

Round 1 explored barriers, enablers, and strategies to address risk and protective factors for burnout in VN/Ts. Results for Round 1, summarising the themes identified among barriers and enablers to address risk and protective factors, as well as the mean rating for how easy or difficult participants thought that the risk or protective factor is to manage, can be seen in Table 1.

Perceived ease of management ratings for all risk and protective factors ranged from 2.38 (±1.01) to 3.74 (±1.03) on a scale of 1 (very difficult) to 5 (very easy). Consensus around ease of management was achieved in only three out of 13 items, with scores for the remaining items spread across at least four out of five ratings. The factor that was rated the most difficult to address in Round 1 was Problem 5 ‘Poor management and leadership of the team’. One participant suggested that “*It is very difficult to address poor management or leadership because that would involve leadership admitting that those things are a problem*”. In contrast, Protective factor 2 ‘Knowledge of having a positive impact on a patient or client’ was rated the easiest protective factor to leverage, with numerous client and feedback systems being proposed that one participant described as “*Pretty easy and low cost!*”.

Analysis of the qualitative data exploring barriers and enablers identified two key overarching themes. First, there is a lack of training and support for leaders as well as few positive role models in this area. One participant noted that this is an ongoing cycle, where “*Leaders in the vet profession don’t routinely get exposed to good leadership so learn poor behaviours, then are promoted into the role with no support*”. In addition, there is a lack of, or variability in, VN/T regulation across all countries represented in the study, which may impact the capacity to effect positive change in several risk areas. One participant highlighted the extent of the impact, noting that “*Out of date/touch regulations and rules with regard to VN/T capabilities that would value add to the business, animal welfare, workload for vets, and job satisfaction for VN/Ts*”. Other frequently cited barriers included workforce shortages, existing levels of burnout in the team or management, hierarchical organisational structures and a lack of workplace resources such as time or money. Enablers included access to technology to support efficiency, professional networks, open and transparent communication, and breadth of skills within the team.

Consistent themes were also identified across the proposed solutions. These focused primarily on addressing leadership issues by improving recruitment procedures for leadership roles, providing ongoing leadership training and support, and utilising management consultants where necessary to address gaps in business expertise. In addition, a number of strategies were proposed to support suggested solutions but were beyond the scope of individual clinics to implement. These included public education campaigns to increase awareness of industry challenges such as workload; the promotion of better understanding and acknowledgment of VN/Ts in professional liability insurance policies; and advocacy initiatives to lobby government and industry bodies for better pay standards, mandatory registration, and title protection.

Specific solutions and strategies to address each risk and protective factor were developed based on common themes that emerged from participant responses. A range of broad solutions were identified for each risk factor, with more specific actions proposed to help achieve this solution. For example, to increase opportunities for skills and knowledge utilisation, one proposed solution was ‘Implement systems to support delegation’. Suggested actions included: implementing VN/T-to-patient and veterinarian ratios, developing mentoring programs, developing clear standard operating procedures (SOPs) for performing clinical tasks, employing support staff to complete non-clinical tasks, and implementing team rounds to support collaboration. Common themes found across all solutions included improving communication, training and support, and workflow systems.

### 3.3. Round 2

Round 2 asked participants to rate the solutions and strategies, developed in Round 1, for ease of implementation (solutions and strategies) and effectiveness in addressing burnout (solutions). Results for Round 2, summarising the proposed solutions developed from the Round 1 responses, as well as the mean and consensus ratings for how easy each solution and strategy would be to implement, and how effective each solution would be in addressing burnout, can be seen in Table 2 and Table 3.

A broad lack of consensus was found regarding the perceived ease of implementation with scores for 50 out of 51 (98%) of the proposed solutions and strategies spread across at least four different ratings on a Likert scale of one (very easy) to five (very difficult). This lack of consensus could be explained by general qualitative data across all risk and protective factors, which suggested that ease of implementation was expected to vary depending on a range of contextual factors in the affected clinic. Existing workplace climate, including team culture, leadership quality, staff willingness for change, attitudes and behaviours of existing staff, and the ability to effectively design and implement change processes, were identified by participants as aspects that impact the complexity of effecting workplace change. Based on these findings, achieving consensus on ease of implementation was determined to be unlikely due to there being too many variables to determine one, or any, best practice recommendations. Advancement of these questions to further rounds was discontinued.

In contrast to ease of implementation, consensus around effectiveness was reached in 39 out of 40 (97.5%) proposed solutions. The majority of solutions (87.5%) were rated as effective/very effective, with three (7.5%) rated as effective/neutral, one (2.5%) rated as ineffective/neutral, and one (2.5%) where consensus was not reached. Qualitative data highlighted three key challenges and considerations that applied broadly across the risk factors and proposed solutions. First, implementation time and strategy must be considered when initiating any change process to set realistic expectations. Solutions that rely on culture change, for example, require long term commitment, with one participant estimating that “*It takes time to engage the team and shift the culture (1–2 years)*”, and another noting that change management processes must be carefully considered “*It needs to be done* with *the people not* to *the people and it needs to be authentic not just words*”. In addition, historical cultural attitudes embedded in the industry also need to shift in order to reduce barriers to change as illustrated by the following response: “*The most difficult part […] is getting past the ‘but I have always done it this way’ mentality*”. One participant suggested that embedding leadership skills in professional training programs could be of benefit “*The changing of workplace wellbeing, culture and leadership could be quite easy if these issues were addressed within [Doctor of Veterinary Medicine] DVM school trainings as well as technician and assistant schools*”. Finally, in some geographical regions, progress on issues including utilisation, progression opportunities, and remuneration, was reported as being hindered by lack of regulation or title protection, with one participant suggesting that “*Registration and accreditation of [VN/Ts] with delineation of duties based on experience and education will assist professional growth and recognition […] and be covered under registration/insurance just as vets are*”. Another participant noted that the lack of, or unclear, regulation impedes veterinarian’s willingness to delegate tasks that VN/Ts are trained to perform “*[Vets] prefer to do things themselves because they know where accountability will land*”.

Out of all risk factors analysed, high workload had the most effective solution: ‘Improving staff retention’, which was rated as considerably more effective than: ‘Hiring more staff’. One participant noted that “*Hiring more staff will always be a problem: 1. As we have a decline in the numbers of both DVMs and VN/Ts interested in serving this field, 2. Until we positively impact culture (which drives retention) and workload management systems*”. Another stated that “*Hiring more staff is most likely not cost effective for many clinics, improving workflow and retention of staff is better*”. High workload also had the least effective solution: ‘Preventative healthcare focus to reduce the number of cases seen’. Participants suggested that this was beyond the capacity of an individual clinic to manage, with one noting that “*The public health promotion to clients and consumers would require a HUGE effort from all sectors of the profession and would be extremely challenging for a clinic to do on their own to change client and consumer behaviours*”.

The most highly rated effective solutions overall addressed poor management and leadership of the team, which had been rated the hardest risk factor to address in Round 2. Participants noted that leaders in the veterinary industry are often not selected for leadership qualities, therefore, early support is needed “*In the veterinary industry (VN/T and vet) the leaders are often those that own the practice or ‘fall into’ the position with length of service or when staff leave. Better or greater focus on leadership and management skills in undergraduate programs would help*”, as well as ongoing peer support “*Leadership peer groups that provide coaching are a much-needed solution*”.

Dealing with clients showing rude and abusive behaviours had the only solution where consensus was not reached: ‘Communicate clear behavioural expectations to clients’. Conflicting opinions around this focused on how the solution is implemented, with support for providing clear expectations for both clients and veterinary staff. One participant noted that “*Expectations from clients alone without what they receive in return may be damaging on first impressions*”, and another confirming “*You can post signs for clients about poor behaviour—what do they care? They can just go someplace else*”. A third participant suggested that “*When a client begins at a practice, [they] should be signing some sort of agreement of what the practice will provide for them and the required client behaviours*”.

Analysis of data from open-ended questions in Round 2 was used to revise the proposed solutions and suggested actions derived from Round 1. This included changes to language to promote inclusive practice. For example, for Problem 3, exploring solutions to a negative team culture, the proposed action: ‘support calling *out* unacceptable behaviour’ was changed to ‘support calling *in* of unacceptable behaviour’. It also included changes to promote a growth mindset, acknowledging that individuals have the capacity to change. For example, in Problem 9, exploring solutions to client incivility, ‘Having to deal with rude or abusive clients’ was changed to ‘Dealing with clients expressing rude or abusive behaviours’. In addition, a solution rated as ineffective/neutral was removed, and proposed timelines to guide implementation of solutions were added based on feedback around setting realistic expectations for implementation. The final recommendations can be seen in Table 4.

Proposed strategies and suggested actions to leverage burnout protective factors were also updated based on analysis of Round 2 data and can be seen in Table 5.

## 4. Discussion

The aim of this study was to develop effective and feasible organisational management strategies to address burnout in VN/Ts. This was achieved by utilising the Delphi method to first identify the barriers and enablers to effecting change and then explore the perceived ease and effectiveness of proposed strategies, to develop recommendations.

### 4.1. Industry-Wide Barriers

The findings revealed that the same barriers exist across all countries involved in this study. Some barriers, such as inadequate regulation and lack of leadership education in veterinary and VN/T training programs, are beyond the capacity of individual clinics to effect change and require industry-wide or government interventions. The lack of, or uncertainty around, regulation of VN/Ts was a key barrier to addressing multiple burnout risk factors. Whilst current regulatory requirements vary between, and within, countries, participants from all represented countries (USA, UK, Australia, Canada, and New Zealand) reported the impact of inadequate regulation on VN/T utilisation. Local associations not permitting VN/Ts to perform skills they are trained and qualified in, as well as poor veterinarian education on VN/T regulations, have been found to contribute to poor utilisation [36,37]. Guidelines for optimal utilisation of VN/Ts, published by the American Animal Hospital Association (AAHA) [38], outline the positive impact on the whole veterinary team, including improved patient care, staff retention, and financial sustainability of the business, and provide multiple resources to support improved utilisation. Further research, however, is required to measure the impact of these guidelines in the future.

Inadequate support for leaders was also found to be a barrier to addressing numerous burnout risk factors. Previous studies have highlighted the important role that leadership plays in managing veterinary team burnout through actions such as support, advocacy, recognition, and empowerment of staff [1,3]. These findings were replicated in the current study, but it was also revealed that many veterinary team leaders lack the necessary training or time to succeed in the role. Promotion of individuals to leadership roles based on clinical expertise, or length of service, was reported as an embedded cultural problem within the industry, resulting in a lack of positive role models or mentors to drive change. Provision of training or support to leaders was proposed as a solution strategy for over half of the risk factors that were reviewed, which illustrates the extent of the issue. Improved recruitment processes for leadership positions, as well as better support and training for those interested in taking on a leadership role, are vital to reducing burnout in VN/Ts as well as VN/T leaders. Furthermore, non-leadership career progression pathways, other than leaving the industry, must be offered to increase options for advanced VN/Ts in order to prevent people advancing to management roles as the only career progression option.

### 4.2. Clinic Specific Barriers

Unique barriers within individual clinics focused on the existing workplace climate and therefore, will vary between clinics. Quality of leadership, existing team culture, staff willingness for change, existing level of staff burnout, financial health of the clinic, and the ability to effectively design and implement change processes, are all issues that must be objectively evaluated before determining suitable strategies for implementation.

Addressing poor leadership (Risk factor 5) was found to be a particular challenge as it requires leaders to accept that they are part of the problem. Whilst this poses a critical barrier to effecting positive change for VN/T teams, qualitative data from this study also acknowledged the risk of burnout in leaders. Research suggests that negative leader well-being is associated with negative employee well-being [39]. However much of the literature focuses on the effects of leadership on employee burnout, with limited research exploring burnout in leaders, despite reports of high levels of stress and demands in their role [39,40]. In addition to providing support to improve leadership skills, it is essential that well-being support is also provided to leaders in order to enable them to perform to the best of their ability. Similarly, implementing significant organisational change has been found to contribute to burnout in teams [41]. Whilst pre-existing burnout in the team does not prevent the introduction of new systems, required levels of support, such as time and resources, should be re-evaluated in order to maximise adaptive reserve and resilience within the team and avoid increased burnout and turnover [42].

Reluctance of staff to engage in a change process due to attitudes such as “we’ve always done it this way”, was identified as a barrier in the current study and has also been reported elsewhere [37,43]. Resistance to organisational change has been reported as one of the key reasons why change initiatives fail [44]. Reasons for resistance include perceived loss of control and lack of fairness [44,45], which comprise two of the six key Areas of Worklife found to contribute to burnout [46]. When encountering resistance to change, it is, therefore, essential to understand the underlying reasons for resistance, in order to address these directly [44].

Clinic-specific barriers that are within the capability of individual leaders to address will vary widely between clinics. Consideration must, therefore, be given to the existing workplace climate before selecting which strategies to implement, and indeed, how to implement them, in order to maximise chances of success.

### 4.3. Solutions

#### 4.3.1. Risk Factors

Common themes among the proposed solutions included improving communication, developing a culture of psychological safety, developing progression pathways for VN/Ts with embedded training and support, developing clear policies in collaboration with the team, and reviewing workplace systems to enhance efficiency. Many of the solutions, such as improving workplace culture, will be a long process requiring time, commitment, and resources to ensure that changes are successful and become embedded in the ongoing normality of the clinic [47]. Kotter’s [48] change management process suggests that short term wins can help to build morale and momentum to help sustain motivation within larger change efforts. It may be possible, and even beneficial, therefore, to implement a combination of short- and long-term strategies simultaneously to help generate positive change in the short term.

Of the ten workplace risk factors evaluated in the study, high workload and lack of support have been found to be significant contributors to burnout in VN/Ts [16]. In the current study, improving staff retention was considered a more effective strategy for reducing workload (Risk factor 1) than hiring more staff. Reasons for VN/T turnover have been found to include poor financial outlook, lack of career progression opportunities, lack of utilisation, poor workplace culture, and lack of employer support [49,50], all of which have also been found to be risk factors for burnout in VN/Ts [16]. Factors predicting retention in veterinary nurses have been described as multi-factorial and individual [50]. The introduction of ‘stay interviews’ was one of the proposed actions to achieve this solution in the current study. These short, proactive discussions between leader and individual team member are conducted at regular intervals and establish what works well in their current role and what can be improved and have been shown to have positive results in human nursing teams [51,52].

Improving workplace culture (Risk factor 3) was identified as particularly challenging to address due to the time it takes to establish culture change within an organisation. Research suggests that culture impacts employee engagement, morale, and cooperation, all of which play a key role in the implementation of successful change initiatives [47]. Proposed solutions focused on breaking down barriers within the team through actions such as developing team vision and values collaboratively, establishing support from the leadership team, and promoting regular and transparent communication. Establishing a need for change and developing a shared vision are proposed to be two key stages of successful change management [53]. Research suggests that acknowledging current problems within the team, developing a clear vision, and combined leadership and employee support are all required for culture change to be successful [47]. Whilst these have all been identified as separate actions within the recommendations, it is, therefore, important to ensure that they are implemented collectively in order to maximise the change of success.

#### 4.3.2. Protective Factors

Factors that protect against burnout in VN/Ts include having control over one’s work, knowledge of having a positive impact, and being included in patient care decision making [16]. As with risk factors, the ability to leverage each of these will vary between clinics, therefore strategies should be carefully considered before implementation. The perceived effectiveness of these strategies was not measured, as protective factors are aimed at preventing the onset of burnout, rather than reducing existing burnout [54]. However, a notable overlap of themes can be seen between proposed primary prevention strategies (protective factor strategies) and secondary prevention strategies (solutions to risk factors). Teamwork, collaboration, improved communication, support, and development opportunities, can be seen across recommendations for both primary and secondary burnout prevention. As such, it may be beneficial for clinic leaders to consider all recommendations—regardless of which burnout risk factors have been identified in their clinic—in order to take a proactive approach to burnout management within their teams.

### 4.4. Implementation of Solutions

Change management is challenging, and organisational change initiatives have been reported to fail in 70% of cases, and in up to 90% of cases addressing culture change [55]. Change is, however, essential for improvement and ultimately, the survival of any organisation [55]. The findings from this study showed that a positive and psychologically safe workplace culture is fundamental to the success of a large number of the proposed solutions. Previous research shows that a solid understanding of the operational process of change, as well as establishing upper management support and awareness of the existing issues, are critical to driving successful change [56]. Instruments to measure organisational culture and health may be of use in conducting impartial evaluations and have been designed for use in human healthcare [57,58]. These surveys conduct a full 360-degree evaluation of areas such as leadership and management effectiveness, staff wellbeing, and patient safety, and provide a valuable reference for developing veterinary focused assessment tools. Use of evaluation tools such as these can help to increase leadership awareness of areas where their perceptions of the organisational climate do not align with those of their teams. This understanding will support leaders to select targeted strategies which are more likely to be successful in addressing existing workplace issues.

In addition to assessing workplace climate, the time and effort involved with strategy implementation must be considered and clearly communicated to the team in order to set realistic expectations for both staff and leaders. Employee attitudes towards change have been found to be a key determinant of its long-term success, therefore any change process—regardless of the intended benefits for staff—must be carefully managed [59]. One of the key barriers to addressing burnout in this study was found to be the lack of leadership or management expertise within the industry. In veterinary clinics where knowledge of workplace program design and implementation is lacking, external support such as the use of business management consultants with an in-depth understanding of the veterinary industry should, therefore, be considered to increase the chances of success.

The dissemination of these and future findings is critical to effecting change. Whilst it is beyond the scope of this study, which focused on implementation of change within the workplace, industry wide change is essential to achieving meaningful progress. Including leadership education in veterinary and VN/T training, improving VN/T regulation, and increasing awareness of burnout reduction strategies across the industry can all make a significant contribution to reducing burnout. In addition, tackling misinformation around topics such as VN/T utilisation, and individuals’ weaknesses leading to burnout, is imperative and may benefit from platforms such as social media to discredit broad misconceptions [60].

Finally, it should be noted that whilst burnout is the result of workplace stressors and, thus, the workplace has a responsibility to address them where possible, not all burnout contributors can be completely controlled or eliminated [3]. There is, therefore, a shared responsibility for VN/Ts to implement individual coping strategies to strengthen the effects of workplace efforts. Whilst these may be implemented by the individual, however, many, for example work-life balance, counselling, and education, may also require, or benefit from, workplace support [3].

### 4.5. Limitations and Further Research

This study had some limitations worth noting. First, a high level of consensus around effectiveness of solutions was achieved in Round 2 with only minor proposed modifications which did not justify additional rounds. As a consequence, there was no opportunity to measure stability of the results over successive rounds. There is significant variance among the accepted closing criteria for Delphi studies. Some authors argue that stability must be measured in conjunction with consensus in order to confirm the validity of results [61]. Others argue that either consensus alone, or a pre-determined number of rounds, is a more commonly used criterion for terminating a Delphi study [62]. As such, additional rounds would have enabled the measurement of stability in addition to consensus, but at the risk of extending the study and further imposing on the goodwill of participants. Further Delphi studies would be beneficial to confirm the reliability and proposed effectiveness of these recommendations. Second, whilst all efforts were made to recruit panel members from a range of representative countries, almost 50% of the panel comprised members from one country (USA), whilst the other 50% represented members from the remaining four countries (UK, Australia, Canada, New Zealand). It is, therefore, possible that some proposed solutions may be more applicable to the culture and legislative climate of the USA. In addition, all panel members were recruited from developed countries and therefore data from developing countries was not captured. The findings, therefore, may not translate to the different challenges that may be experienced by VN/T leaders in developing countries and further research would be beneficial to explore the generalisability of these recommendations. Finally, gaining expert opinion fills a gap in existing knowledge on effective organisational strategies to address burnout in VN/Ts. However, further research is required to evaluate and measure the effectiveness of proposed interventions in order to know if the recommendations are actually effective in preventing burnout.

## 5. Conclusions

Burnout in VN/Ts has negative impacts on the individual, patient, and business, but existing research lacks clear recommendations on workplace strategies to support better management of burnout at the source. This study developed 39 expert-led workplace burnout reduction strategies and recommended actions to guide VN/T managers worldwide in addressing 10 known risk factors for burnout in VN/Ts. To the authors’ knowledge, this is the first research to explore expert recommendations to address burnout in veterinary or animal care professionals. Whilst some of the recommendations are uniquely relevant to VN/Ts, many apply across broader animal care professional roles and may therefore help to guide leaders in addressing burnout more widely.

Proposed strategies to address burnout, and associated recommended actions, are specific to each risk factor, but many had similar overarching themes. These included providing support and education to leaders, improving communication, fostering a culture of psychological safety, developing clear guidelines and protocols in collaboration with the team, providing clear progression pathways, and supporting delegation of tasks to VN/Ts, as well as redistributing tasks for which they are overqualified. Successful implementation of strategies relies on first, identifying the presence of workplace risk factors through assessment of burnout among staff, and then, evaluating the existing organisational climate, in order to select the most appropriate strategies. A good understanding of change processes will also help to maximise success. In addition, the study identified the need for broader change initiatives, including development of clearer regulation of VN/Ts, and inclusion of leadership education within veterinary and VN/T training programs, to better support veterinary clinics in achieving positive change within the industry. These findings provide practical guidelines to help veterinary clinics reduce the risk of burnout in VN/Ts, as well as insights for consideration by the broader veterinary industry to support professional growth and a more sustainable future.

## Figures and Tables

**Table 1:** Barriers and enablers to addressing risk and protective factors associated with burnout in VN/Ts derived from Round 1.

Risk Factor	Barriers to Addressing the Risk Factor Derived from Content Analysis Themes	Enablers to Addressing the Risk Factor Derived from Content Analysis Themes	Perceived Ease of Management Rating (Mean/5.00 (*SD*))	Consensus Rating (Participant Agreement %)
1. The workload is too high	Financial constraints of the business Unpredictable workload Workforce shortages and existing levels of burnout Lack of clear systems and procedures to guide workflow Lack of teamwork and communication	Necessity forcing clinics to address workflow systems Collaborative leadership culture Technologies that support workload streamlining and efficiencies Flexible scheduling	2.66/5.00 (±0.79)	Consensus: Difficult/neither difficult nor easy (82.93%)
2. There is a lack of opportunities to utilise skills and knowledge for which veterinary nurses/technicians are trained and qualified	Understaffing leading to a lack of time and support Lack of trust from veterinarians Resistance to change Small clinic size or staff hierarchies	Professional networks and community support Broad team skillset for mentorship Veterinarian awareness of VN/T education and capabilities Leadership advocacy	3.00/5.00 (±1.14)	Consensus not reached
3. A negative team culture exists (for example: bullying, gossiping, criticism, or general incivility)	Lack of leadership awareness or accountability Lack of psychological safety Fear of losing staff Industry workforce characteristics (female/mental health/generational differences)	Industry awareness and education initiatives (e.g., Veterinary Incivility Toolkit) Budget for resources and training Regular communication and opportunities for feedback	2.52/5.00 (±0.96)	Consensus not reached
4. There is a lack of, or unclear communication from both management and within the team	Large clinic or multiple sites Variance in communication styles and preferences Time limitations Organisational hierarchies and multi-level communication	Culture of psychological safety Regularly scheduled meetings or discussion forums Access to communication platforms such as Teams or Slack Structured and consistent communication channels	3.23/5.00 (±1.07)	Consensus not reached
5. There is poor management/leadership of the team (for example: micromanagement, favouritism, lack of support, or lack of action on team conflict)	Poor leadership training or support Poor leadership role models Negative workplace culture Lack of reporting or feedback mechanisms for staff	Access to leadership training and resources Access to leadership mentoring and professional networks Shallow organisational hierarchies Open communication and feedback channels between team and leaders	2.38/5.00 (±1.01)	Consensus not reached
6. There is an expectation of working overtime, not having a break, and a general lack of flexibility in rostering	Industry cultural norms around prioritising patients over staff wellbeing Workforce shortages Unpredictable workload Resistance to change	Access to rostering software Legislation around breaks requirements and maximum shift length (where applicable) Consistent messaging and advocacy Wellness resources and initiatives	2.86/5.00 (±0.92)	Consensus not reached
7. Remuneration is poor	Lack of business expertise Community attitudes to veterinary fees Financial limitations or of the business Lack of industry regulation or clear professional structure	Workforce shortage driving wages up Appropriate utilisation of VN/Ts and support staff to reduce costs Professional advocacy and legislative changes (where applicable) Management business acumen	2.48/5.00 (±0.99)	Consensus not reached
8. There is a lack of opportunity for progression or development	Lack of clinic resources (monetary and staffing) Small clinics lacking need for advanced roles Lack of industry awareness of VN/T value or potential Fear of change from veterinarians and leadership	Abundance of available CE and educational resources Innovation and opportunities for lateral progression Leadership awareness of VN/T capabilities and value Broad team skillset facilitating mentorship and coaching	2.79/5.00 (±0.86)	Consensus: Difficult/neither difficult nor easy (80.65%)
9. Having to deal with rude or abusive clients	Fear of repercussions (client loss/negative reviews/litigation) Lack of training in communication and de-escalation “The customer is always right” mentality Lack of resources (time/staffing/support) to adequately manage situations	Positive and well-developed client relationships Dedicated client support roles (e.g., veterinary social workers) Availability of training and resources on communication Access to support (peer networks, Employee Assistance Program)	2.86/5.00 (±1.13)	Consensus: Difficult/Neither difficult nor easy (97.74%)
10. There is a lack of appreciation, feeling valued, or being heard, by management	Diversity in staff acknowledgement needs and preferences Burned out managers Negative or hierarchical workplace cultures Poor management role models and lack of training	Professional advocacy efforts on VN/T appreciation Abundance of existing recognition programs to model Workplace cultures that support open communication and feedback to management	2.86/5.00 (±1.27)	Consensus not reached
**Protective Factor**	**Barriers to Leveraging the Protective Factor Derived from Content Analysis Themes**	**Enablers to Leveraging the Protective Factor Derived from Content Analysis Themes**	**Perceived Ease of Leverage Rating (Mean/5.00 (*SD*))**	**Consensus Rating (Participant Agreement %)**
1. Having some control over the schedule or expected tasks	Hierarchical or negative workplace cultures High and unpredictable workloads Staffing shortages Conflicting team member needs or skill levels	Flexible staffing models Collaborative team culture Cross trained staff to increase task capabilities	3.09/5.00 (±0.89)	Consensus not reached
2. Knowledge of having a positive impact on a patient or client	Shift work rosters—absence at patient discharge Lack of client awareness of VN/T role in patient care Absence of feedback systems Negativity bias—natural focus on negative outcomes	Ability to provide high quality care Room to build connections with clients Clear and established client, and internal, feedback systems	3.74/5.00 (±1.03)	Consensus not reached
3. Being trusted with, and involved in, decisions around patient care	Hierarchical or negative workplace culture Negative veterinarian attitudes towards VN/T capabilities Lack of veterinarian experience and/or self-confidence Lack of, or unclear, professional regulation	Well-developed veterinarian-VN/T relationships Culture of shared team values and collaboration Good patient management record systems	3.13/5.00 (±0.98)	Consensus not reached

**Table 2:** Mean perceived ease of implementation ratings and participant consensus ratings for solutions and strategies to workplace burnout risk and protective factors derived from Round 2.

Risk Factor	Solution	Perceived Ease of Implementation Rating (Mean/5.00 (*SD*))	Consensus Rating (Participant Agreement %)
1. The workload is too high	Hire more staff	2.23/5.00 (±0.57)	Consensus: Difficult/neither easy nor difficult (96.33%)
	Improve staff retention	3.20/5.00 (±1.03)	Consensus not reached
	Implement workload management systems to enhance efficiency and communications around workload issues	3.60/5.00 (±1.07)	Consensus not reached
	Preventative healthcare focus to reduce the number of cases seen	2.62/5.00 (±1.06)	Consensus not reached
2. There is a lack of opportunities to utilise skills and knowledge for which veterinary nurses/technicians are trained and qualified	Provide role clarity on skill level and task expectations of VN/Ts and veterinarians	3.80/5.00 (±1.19)	Consensus not reached
	Support veterinarians to delegate tasks to VN/Ts	3.30/5.00 (±1.06)	Consensus not reached
	Create opportunities for skill utilisation by reducing non-clinical workload and increasing clinical work opportunities	3.30/5.00 (±1.02)	Consensus not reached
	Implement systems to support delegation	3.33/5.00 (±1.12)	Consensus not reached
3. A negative team culture exists (for example: bullying, gossiping, criticism, or general incivility)	Zero-tolerance to incivility at all levels of the workplace	2.67/5.00 (±1.24)	Consensus not reached
	Promote culture change	3.17/5.00 (±1.12)	Consensus not reached
	Set clear expectations on expected behaviour	3.63/5.00 (±1.22)	Consensus not reached
	Provide staff and leadership training and support	3.97/5.00 (±1.0)	Consensus: Easy/Very easy (76.66%)
4. There is a lack of, or unclear, communication from both management and within the team	Increase communication opportunities	3.86/5.00 (±1.01)	Consensus not reached
	Promote and reward good communication	3.57/5.00 (±1.03)	Consensus not reached
	Develop clear communication protocols and reporting lines	3.89/5.00 (±0.96)	Consensus not reached
	Utilise different communication methods	3.64/5.00 (±1.10)	Consensus not reached
5. There is poor management/leadership of the team (for example: micromanagement, favouritism, lack of support, or lack of action on team conflict)	Improve leadership recruitment and training processes	2.67/5.00 (±1.06)	Consensus not reached
	Implement systems to support leaders	3.00/5.00 (±1.02)	Consensus not reached
	Improve workplace communication	3.50/5.00 (±1.01)	Consensus not reached
	Initiate leadership reviews and accountability	3.53/5.00 (±1.04)	Consensus not reached
6. There is an expectation of working overtime, not having a break, and a general lack of flexibility in rostering	Review and adjust staffing to meet clinic needs	2.47/5.00 (±1.04)	Consensus not reached
	Implement clear break and overtime policies	3.23/5.00 (±1.10)	Consensus not reached
	Provide leadership and team training	3.33/5.00 (±1.15)	Consensus not reached
	Review and implement workflow systems to streamline tasks and develop contingency plans	3.00/5.00 (±0.95)	Consensus not reached
7. Remuneration is poor	Offer non-monetary remuneration	2.63/5.00 (±1.19)	Consensus not reached
	Explore opportunities to increase revenue	3.23/5.00 (±1.14)	Consensus not reached
	Implement salary banding and progression pathways	3.60/5.00 (±1.28)	Consensus not reached
	Implement work processes to reduce costs	3.17/5.00 (±1.18)	Consensus not reached
8. There is a lack of opportunity for progression or development	Develop clear progression pathways for VN/Ts	3.33/5.00 (±1.12)	Consensus not reached
	Explore professional growth opportunities	3.43/5.00 (±1.17)	Consensus not reached
	Provide internal VN/T training and support	3.30/5.00 (±1.12)	Consensus not reached
	Promote external VN/T training and support	3.47/5.00 (±1.17)	Consensus not reached
9. Having to deal with rude or abusive clients	Provide clear expectations on client conflict management and empower the team	3.73/5.00 (±1.05)	Consensus not reached
	Create workplace support systems for VN/Ts faced with client abuse	3.40/5.00 (±1.00)	Consensus not reached
	Prepare and train the team for conflict situations	3.53/5.00 (±0.97)	Consensus not reached
	Communicate clear behavioural expectations to clients	3.93/5.00 (±0.83)	Consensus: Easy/very easy (76.66%)
10. There is a lack of appreciation, feeling valued, or being heard, by management	Implement VN/T recognition systems	3.70/5.00 (±0.99)	Consensus not reached
	Increase communication channels between management and VN/Ts	3.67/5.00 (±0.96)	Consensus not reached
	Provide support and training for leaders	3.79/5.00 (±1.05)	Consensus not reached
	Identify what appreciation looks like for individuals	3.90/5.00 (±0.92)	Consensus not reached
**Protective Factor**	**Promotion Strategy**	**Perceived Ease of Implementation Rating (Mean/5.00 (*SD*))**	**Consensus Rating (Participant Agreement %)**
1. Having some control over the schedule or expected tasks	Adopt a collaborative team scheduling approach	3.37/5.00 (±1.10)	Consensus not reached
	Upskill and cross-train VN/Ts	3.07/5.00 (±1.08)	Consensus not reached
	Develop clear expectations for VN/T tasks and roles	3.93/5.00 (±0.83)	Consensus: Easy/very easy (76.66%)
	Implement work systems to enhance clarity and communications around scheduling	3.53/5.00 (±1.04)	Consensus not reached
2. Knowledge of having a positive impact on a patient or client	Implement peer feedback systems	4.16/5.00 (±0.80)	Consensus: Easy/Very easy (84%)
	Encourage clear and open communication within the team	3.37/5.00 (±1.25)	Consensus not reached
	Provide recognition based on individual VN/T needs	3.69/5.00 (±0.97)	Consensus not reached
3. Being trusted with, and involved in, decisions around patient care	Increase veterinarian and leadership awareness of VN/T training and capabilities	3.27/5.00 (±1.11)	Consensus not reached
	Develop clear VN/T capability and advancement levels	3.47/5.00 (±1.11)	Consensus not reached
	Build veterinarian trust in VN/Ts through fostering a collaborative culture	3.07/5.00 (±1.14)	Consensus not reached
	Support VN/T professional development and learning	3.67/5.00 (±1.06)	Consensus not reached

**Table 3:** Mean perceived effectiveness ratings and participant consensus ratings for solutions to workplace burnout risk factors derived from Round 2.

Risk Factor	Solution	Perceived Effectiveness Rating (Mean/5.00 (*SD*))	Consensus Rating (Participant Agreement %)
1. The workload is too high	Hire more staff	3.86/5.00 (±0.85)	Effective/very effective (79%)
	Improve staff retention	4.57/5.00 (±0.74)	Effective/very effective (93%)
	Implement workload management systems to enhance efficiency and communications around workload issues	4.18/5.00 (±0.55)	Effective/very effective (93%)
	Preventative healthcare focus to reduce the number of cases seen	2.75/5.00 (±0.84)	Ineffective/neutral (75%)
2. There is a lack of opportunities to utilise skills and knowledge for which veterinary nurses/technicians are trained and qualified	Provide role clarity on skill level and task expectations of VN/Ts and veterinarians	4.21/5.00 (±0.57)	Effective/very effective (93%)
	Support veterinarians to delegate tasks to VN/Ts	4.25/5.00 (±0.70)	Effective/very effective (93%)
	Create opportunities for skill utilisation by reducing non-clinical workload and increasing clinical work opportunities	4.39/5.00 (±0.74)	Effective/very effective (93%)
	Implement systems to support delegation	4.29/5.00 (±0.71)	Effective/very effective (93%)
3. A negative team culture exists (for example: bullying, gossiping, criticism, or general incivility)	Zero-tolerance to incivility at all levels of the workplace	4.14/5.00 (±0.79)	Effective/very effective (83%)
	Promote culture change	4.38/5.00 (±0.68)	Effective/very effective (90%)
	Set clear expectations on expected behaviour	4.14/5.00 (±0.74)	Effective/very effective (86%)
	Provide staff and leadership training and support	4.28/5.00 (±0.65)	Effective/very effective (90%)
4. There is a lack of, or unclear, communication from both management and within the team	Increase communication opportunities	4.23/5.00 (±0.71)	Effective/very effective (85%)
	Promote and reward good communication	4.08/5.00 (±0.63)	Effective/very effective (85%)
	Develop clear communication protocols and reporting lines	4.12/5.00 (±0.65)	Effective/very effective (85%)
	Utilise different communication methods	3.96/5.00 (±0.6)	Effective/very effective (88%)
5. There is poor management/leadership of the team (for example: micromanagement, favouritism, lack of support, or lack of action on team conflict)	Improve leadership recruitment and training processes	4.38/5.00 (±0.62)	Effective/very effective (93%)
	Implement systems to support leaders	4.31/5.00 (±0.54)	Effective/very effective (97%)
	Improve workplace communication	4.45/5.00 (±0.51)	Effective/very effective (100%)
	Initiate leadership reviews and accountability	4.24/5.00 (±0.74)	Effective/very effective (90%)
6. There is an expectation of working overtime, not having a break, and a general lack of flexibility in rostering	Review and adjust staffing to meet clinic needs	4.17/5.00 (±0.85)	Effective/very effective (79%)
	Implement clear break and overtime policies	4.00/5.00 (±0.89)	Effective/very effective (76%)
	Provide leadership and team training	4.21/5.00 (±0.41)	Effective/very effective (100%)
	Review and implement workflow systems to streamline tasks and develop contingency plans	4.00/5.00 (±0.60)	Effective/very effective (83%)
7. Remuneration is poor	Offer non-monetary remuneration	3.48/5.00 (±0.95)	Effective/ neutral (76%)
	Explore opportunities to increase revenue	3.79/5.00 (±0.62)	Effective/neutral (90%)
	Implement salary banding and progression pathways	4.17/5.00 (±0.6)	Effective/very effective (90%)
	Implement work processes to reduce costs	4.17/5.00 (±0.66)	Effective/very effective (89%)
8. There is a lack of opportunity for progression or development	Develop clear progression pathways for VN/Ts	4.21/5.00 (±0.73)	Effective/very effective (83%)
	Explore professional growth opportunities	4.10/5.00 (±0.56)	Effective/very effective (90%)
	Provide internal VN/T training and support	4.28/5.00 (±0.53)	Effective/very effective (97%)
	Promote external VN/T training and support	4.03/5.00 (±0.68)	Effective/very effective (79%)
9. Having to deal with rude or abusive clients	Provide clear expectations on client conflict management and empower the team	4.17/5.00 (±1.00)	Effective/very effective (83%)
	Create workplace support systems for VN/Ts faced with client abuse	4.10/5.00 (±0.77)	Effective/very effective (83%)
	Prepare and train the team for conflict situations	4.14/5.00 (±0.74)	Effective/very effective (79%)
	Communicate clear behavioural expectations to clients	3.72/5.00 (±1.19)	Consensus not reached
10. There is a lack of appreciation, feeling valued, or being heard, by management	Implement VN/T recognition systems	3.69/5.00 (±0.85)	Effective/neutral (76%)
	Increase communication channels between management and VN/Ts	4.03/5.00 (±0.63)	Effective/very effective (83%)
	Provide support and training for leaders	4.15/5.00 (±0.6)	Effective/very effective (89%)
	Identify what appreciation looks like for individuals	4.45/5.00 (±0.69)	Effective/very effective (90%)

**Table 4:** Proposed solutions and associated actions to address ten workplace burnout risk factors in VN/Ts, ranked in order of effectiveness as rated by Delphi panel members.

**Risk factor 1: The workload is too high**		
**Proposed solutions**	**Effectiveness rating** (mean/5.00 (*SD*))	**Examples of actions that may be implemented include**
Improve staff retention	Very high (4.57/5.00 (±0.74))	**Short term:** Implement clear job goal and progression tracking; improve leader-staff communication; implement exit and stay interviews
		**Medium term:** Improve workplace wellbeing; improve leader responsiveness; empower staff to have a voice; provide leadership and team training on psychosocial hazards and occupational health and safety (OHS) responsibilities
		**Long term:** Improve workplace culture; provide training and support to improve leadership skills; provide staff training and increase VN/T utilisation
Implement workload management systems to enhance efficiency and communications around workload issues	Very high (4.18/5.00 (±0.55))	**Short term:** Develop clear policies and standard operating procedures (SOPs) to provide guidance on caseload and booking protocols; hold regular team meetings; review current systems to improve workflow
Hire more staff	High (3.86/5.00 (±0.85))	**Short term:** Determine skill gaps and capacity to support training of unskilled VN/Ts; hire more VN/Ts and/or non-VN/T support staff to reduce non-clinical workload—level of experience should be determined by needs assessment
		**Medium term:** Implement robust induction and training processes
**Risk factor 2:** **There is a lack of opportunities to utilise skills and knowledge for which veterinary nurses/technicians are trained and qualified**		
**Proposed solutions**	**Effectiveness rating** (mean/5.00 (*SD*))	**Examples of actions that may be implemented include:**
Create opportunities for skill utilisation by reducing non-clinical workload and increasing clinical work opportunities	Very high (4.39/5.00 (±0.74))	**Short term:** Introduce VN/T clinics
		**Medium term:** Utilise technology to manage non-clinical workload—ensuring support is provided; implement progression pathways for VN/Ts
Implement systems to support delegation	Very high (4.29/5.00 (±0.71))	**Short term:** Implement VN/T-to-patient and veterinarian ratios; develop clear SOPs for clinical tasks; introduce team rounds to promote collaboration and increase veterinarian awareness and trust of VN/T capabilities
		**Medium term:** Implement mentoring programs; hire/utilise non-VN/T support staff for non-clinical tasks
Support veterinarians to delegate tasks to VN/Ts	Very high (4.25/5.00 (±0.70))	**Short term:** Provide support from leadership
		**Medium term:** Provide veterinarian training on VN/T education and relevant legislation/regulations; provide delegation training
Provide role clarity on skill level and task expectations of VN/Ts and veterinarians	Very high (4.21/5.00 (±0.57))	**Short term:** Develop clear SOPs outlining veterinary and VN/T tasks and responsibilities—specific to experience/specialisation
		**Medium term:** Introduce VN/T levelling system based on education and experience
**Risk factor 3:** **A negative team culture exists (for example: bullying, gossiping, criticism, or general incivility)**		
**Proposed solutions**	**Effectiveness rating** (mean/5.00 (*SD*))	**Examples of actions that may be implemented include:**
Promote culture change	Very high (4.38/5.00 (±0.68))	**Short term:** Develop team vision and values collaboratively; provide support from leadership; conduct anonymous surveys to measure staff perceptions of culture and identify issues
		**Medium term:** Introduce culture officer role; promote regular and transparent communication; support low level resolution between individuals; leadership team to model positive culture
		**Long term:** Promote psychological safety
Provide staff and leadership training and support	Very high (4.28/5.00 (±0.65))	**Medium term:** Provide dedicated time and training for leadership and staff on areas such as communication skills, conflict management, mental health first aid, civility training, human resources management, diversity, equity and inclusion (DEI) awareness, local employment laws
Set clear expectations on expected behaviour	Very high (4.14/5.00 (±0.74))	**Short term:** Develop clear policies and definitions of bullying; introduce a code of conduct
		**Medium term:** Implement performance improvement plans; implement disciplinary procedures
Zero-tolerance to incivility at all levels of the workplace	Very high (4.14/5.00 (±0.79))	**Short term:** Identify causes of incivility and address issues early; apply zero tolerance policies equitably across all roles
		**Medium term:** Support calling in unacceptable behaviour; implement disciplinary procedures and termination if necessary
**Risk factor 4:** **There is a lack of, or unclear, communication from both management and within the team**		
**Proposed solutions**	**Effectiveness rating** (mean/5.00 (*SD*))	**Examples of actions that may be implemented include:**
Increase communication opportunities	Very high (4.23/5.00 (±0.71))	**Short term:** Introduce interdisciplinary and individual team meetings; implement team huddles and debriefs at the start and end of the day; implement a leadership open door policy; create feedback opportunities
Develop clear communication protocols and reporting lines	Very high (4.12/5.00 (±0.65))	**Short term:** Implement clear reporting lines; establish a single source of information; develop communication templates; ensure availability of recordings or minutes for those unable to attend; provide training in new protocols and systems
Promote and reward good communication	Very high 4.08/5.00 (±0.63))	**Medium term:** Promote leadership modelling of good communication; provide communication training; celebrate effective communication
		**Long term:** Promote psychological safety
Utilise different communication methods	High (3.96/5.00 (±0.60))	**Short term:** Seek input on individual preferences for communication; consider personality types, e.g., introverts and adapt bidirectional communication accordingly; implement anonymous and identified feedback systems
		**Medium term:** Utilise technology to increase accessibility—ensuring support is provided; implement a centralised communication hub
**Risk factor 5:** **There is poor management/leadership of the team (for example: micromanagement, favouritism, lack of support, or lack of action on team conflict)**		
**Proposed solutions**	**Effectiveness rating** (mean/5.00 (*SD*))	**Examples of actions that may be implemented include:**
Improve workplace communication	Very high (4.45/5.00 (±0.51))	**Short term:** Implement feedback systems; hold regular team meetings; promote transparency
		**Long term:** Promote psychological safety
Improve leadership recruitment and training processes	Very high (4.38/5.00 (±0.62))	**Short term:** Adopt recruitment strategies focused on leadership and clinical skills/experience not just clinical seniority
		**Medium term:** Implement succession planning
		**Long term:** Provide ongoing training in leadership and human resource management
Implement systems to support leaders	Very high (4.31/5.00 (±0.54))	**Short term:** Promote empowerment and support from upper management; Promote role modelling and introduce mentorship programs
		**Medium term:** Implement allocated and protected admin time for those in mixed leadership/clinical roles; implement management levelling system to support development
Initiate leadership reviews and accountability	Very high (4.24/5.00 (±0.74))	**Short term:** Develop and communicate clear expectations; implement structured leadership review systems such as 360 reviews
		**Medium term:** Implement performance management processes
**Risk factor 6:** **There is an expectation of working overtime, not having a break, and a general lack of flexibility in rostering**		
**Proposed solutions**	**Effectiveness rating** (mean/5.00 (*SD*))	**Examples of actions that may be implemented include:**
Provide leadership and team training	Very high (4.21/5.00 (±0.41))	**Medium term:** Provide dedicated time for team training on areas such as communication and teamwork, wellness, workplace legislation; provide dedicated time for leadership training on turnover costs and effective workload management; implement cross-training of team skills to enable greater support
Regularly review and adjust staffing to meet clinic needs	Very high (4.17/5.00 (±0.85))	**Short term:** Implement an on-call per diem roster in the event overtime is required
		**Medium term:** Perform a needs analysis to determine gaps; hire more VN/Ts and/or non-VN/T support staff to meet workload, including flexible or part time roles to increase coverage at busy periods and float VN/Ts to cover breaks
Review and implement workflow systems to streamline tasks and develop contingency plans	Very high (4.00/5.00 (±0.60))	**Short term:** Implement limited service/bypass protocols (seeing only critical patients and redirecting non-critical cases elsewhere); develop clear policies to prevent overbooking; hold morning team huddles to plan workflow; utilise rostering software to assist; utilise telemedicine and triaging support services
		**Medium term:** Develop team pipelines to improve workflow; partner with local clinics to share relief or floating VN/Ts
Implement clear break and overtime policies	Very high (4.00/5.00 (±0.89))	**Short term:** Introduce scheduled break times, or break guidelines to be coordinated by shift lead; implement overtime bonus pay structures
		**Medium term:** Cultivate a casual VN/T bank for last minute support
**Risk factor 7:** **Remuneration is poor**		
**Proposed solutions**	**Effectiveness rating** (mean/5.00 (*SD*))	**Examples of actions that may be implemented include:**
Implement salary banding and progression pathways	Very high (4.17/5.00 (±0.60))	**Short term:** Develop clear job descriptions and skill levels—ensuring growth is not limited to title; promote transparency around salary banding as well as clinic running costs
		**Medium term:** Implement pay commensurate with skill level; develop progression pathways and support to increase responsibility and skill level
Implement work processes to reduce costs	Very high (4.17/5.00 (±0.66))	**Medium term:** Utilise technology to automate routine tasks—ensuring support is provided; implement appropriate utilisation of VN/Ts to increase veterinarian capacity
Explore opportunities to increase revenue	High (3.79/5.00 (±0.62))	**Short term:** Ensure correct charging; increase prices; promote client education on pet insurance
		**Medium term:** Introduce new services (e.g., VN/T clinics, telehealth); conduct budget forecasting
Offer non-monetary remuneration	High (3.48/5.00 (±0.95))	**Short term:** Develop a structured and equitable system
		**Medium term:** Determine individualised options in consultation with staff members as suited to their needs (e.g., childcare support, travel vouchers, time off for study)
**Risk factor 8:** **There is a lack of opportunity for progression or development**		
**Proposed solutions**	**Effectiveness rating** (mean/5.00 (*SD*))	**Examples of actions that may be implemented include:**
Provide internal VN/T training and support	Very high (4.28/5.00 (±0.53))	**Short term:** Implement individual development plans; support time off for study
		**Medium term:** Implement a development and training officer role; provide leadership training; develop mentoring/coaching programs
Develop clear progression pathways for VN/Ts	Very high (4.21/5.00 (±0.73))	**Short term:** Develop clear progression bands; develop clear VN/T job descriptions with training to support growth within the clinic
		**Medium term:** Support both leadership and non-leadership progression pathways; implement growth lattices—supporting both vertical and horizontal development
Explore professional growth opportunities	Very high (4.10/5.00 (±0.56))	**Short term:** Identify areas for increased responsibility
		**Medium term:** Support undertaking specialised qualifications that can be utilised by the clinic; develop specific roles that match individuals’ interests or skills
Promote external VN/T training and support	Very high (4.03/5.00 (±0.68))	**Short term:** Identify networking opportunities; signpost funding opportunities and access to CE; identify and approach other industry members for mentoring (e.g., VTS)
		**Medium term:** Establish a continuing education funding program with requirement for funding recipients to share new knowledge with the team
**Risk factor 9: Dealing with clients expressing** **rude or abusive behaviours**		
**Proposed solutions**	**Effectiveness rating** (mean/5.00 (*SD*))	**Examples of actions that may be implemented include:**
Provide clear expectations on client conflict management and empower the team	Very high (4.17/5.00 (±1.00))	**Short term:** Promote zero tolerance for abuse of staff; develop clear guidelines on what constitutes acceptable and non-acceptable behaviour; develop guidelines and provide training on when to escalate issues; develop and provide training on communication protocols for conflict situations
Prepare and train the team for conflict situations	Very high (4.14/5.00 (±0.74))	**Short term:** Identify and address issues that contribute to poor client experience; ensure staff are never rostered alone; install security systems (e.g., CCTV, duress alarms); develop client information on external financial and emotional support services
		**Medium term:** Provide regular staff training in de-escalation and emotional intelligence
Create workplace support systems for VN/Ts faced with client abuse	Very high (4.10/5.00 (±0.77))	**Short term:** Provide —and ensure access to—leadership support; implement debriefs after difficult interactions; Implement reporting systems for incidents
		**Medium term:** Employ social workers in the team with training to manage and support distressed clients
		**Long term:** Promote teamwork and support through improving workplace culture—refer to risk factor 3
Communicate clear behavioural expectations to clients	High (3.72/5.00 (±1.19))	**Short term:** Develop code of conduct contracts for new and/or existing clients outlining two-way expectations of the client-clinic relationship; introduce waiting room signage; provide clear and transparent information on costs and wait times; outline the consequences of abusing staff
**Risk factor 10:** **There is a lack of appreciation, feeling valued, or being heard, by management**		
**Proposed solutions**	**Effectiveness rating** (mean/5.00 (*SD*))	**Examples of actions that may be implemented include:**
Identify what appreciation looks like for individuals	Very high (4.45/5.00 (±0.69))	**Short term:** Survey the team; implement individual development plans; Include individual motivator discussion as part of new staff inductions
		**Medium term:** Provide dedicated time for leaders to spend with staff
Provide support and training for leaders	Very high (4.15/5.00 (±0.60))	**Medium term:** Provide training on areas such as effective communication, emotional intelligence, diversity, equity and inclusion (DEI), culture, intrinsic motivators; develop mentoring and peer group programs
Increase communication channels between management and VN/Ts	Very high (4.03/5.00 (±0.63))	**Short term:** Implement feedback systems; hold regular meetings; establish preferred communication channels in consultation with the team; Implement open door policies
Implement VN/T recognition systems	High (3.69/5.00 (±0.85))	**Short term:** Introduce initiatives in collaboration with the team such as newsletters, VN/T of the month, long service awards, staff appreciation days; include recognition as a standing agenda item in meetings
		**Medium term:** Establish a staff appreciation committee; establish a staff appreciation fund
**Effectiveness rating scored out of 5:** Very low = 0–1; Low = 1.1–2; Neutral = 2.1–3; High = 3.1–4; Very high = 4.1–5 **Duration of implementation:** Short term = 0–6 months; Medium term = 6–12 months; Long term = 1–2 years+ **Note**: Success of many of these strategies relies on effective planning and implementation. If existing expertise is not available within the clinic, the use of—or communication with—external consultants is suggested to increase chance of success.		

**Table 5:** Proposed strategies and associated actions to promote three burnout protective factors in VN/Ts.

**Protective Factor 1: Having some control over the schedule or expected tasks**	
**Proposed strategies**	**Examples of actions that may be implemented include:**
Adopt a collaborative team scheduling approach	VN/T representation in management decision making; morning team huddles to plan tasks, breaks, and teamwork; support innovation to improve scheduling or workflow practices
Upskill and cross-train VN/Ts	Increase capacity to perform in all VN/T roles; build confidence to work autonomously; cross-train to increase opportunity to swap shifts
Develop clear expectations for VN/T tasks and roles	Collaborative development of VN/T shift descriptions with tasks and skill levels outlined
Implement work systems to enhance clarity and communications around scheduling	Clear booking policies to prevent overbooking; break and overtime policies; seek VN/T feedback on scheduling issues and collaboratively problem solve
**Protective Factor 2: Knowledge of having a positive impact on a patient or client**	
**Proposed strategies**	**Examples of actions that may be implemented include:**
Implement peer feedback systems	Kudos boards; team meeting shout outs; communication systems to pass feedback up to management; make acknowledgement part of team huddles and debriefs
Encourage clear and open communication within the team	Improve culture around good communication; leadership modelling of good communication; create regular and easy feedback systems and habits
Provide recognition based on individual VN/T needs	Survey the team to determine preferences; public vs. private feedback; increase team and leadership awareness of generational and career stage influences on individual needs
**Protective Factor 3: Being trusted with, and involved in, decisions around patient care**	
**Proposed strategies**	**Examples of actions that may be implemented include:**
Increase veterinarian and leadership awareness of VN/T training and capabilities	Encourage active involvement of VN/Ts in case discussions providing an opportunity to demonstrate knowledge; education of veterinarians and leaders on VN/T education levels and professional regulation
Develop clear VN/T capability and advancement levels	Clear guidelines around skills and abilities expected at each level; conduct VN/T competency assessments to progress to next level; develop a spectrum of care chart to clearly outline clinical expectations at each level
Build veterinarian trust in VN/Ts through fostering a collaborative culture	Psychological safety; all team patient rounds; veterinarian to VN/T mentoring and reverse mentoring programs; encourage questions and discussion for learning; team debriefing; leadership support; team consulting; collaborative patient care plans
Support VN/T professional development and learning	Individual development plans; in house training; journal clubs; shadowing shifts with experienced VN/Ts; provide clear patient parameter ranges and alert guidelines to inexperienced VN/Ts

## Data Availability

Data are unavailable to share due to privacy restrictions.

## Supplementary Materials

The following supporting information can be downloaded at: https://www.mdpi.com/article/10.3390/ani15091257/s1, Supplementary Materials S1: Delphi study Survey—Round 1; Supplementary Materials S2: Delphi study Survey—Round 2.

## Abbreviations

The following abbreviations are used in this manuscript:

AAHA	American Animal Hospital Association
DVM	Doctor of Veterinary Medicine
ILO	International Labour Organisation
OHS	Occupational Health and Safety
SOPs	Standard Operating Procedures
UK	United Kingdom
USA	United States of America
VN/T	Veterinary nurse and technician
WHO	World Health Organisation
