# Supplementary material for: Workplace Strategies to Reduce Burnout in Veterinary Nurses and Technicians: A Delphi Study
Source: Animals (Basel). 2025 Apr 29;15(9):1257. doi: 10.3390/ani15091257 (PMC12071012; doi:10.3390/ani15091257)
Supplement: Supplementary file 1 [file animals-15-01257-s001.zip › Supplementary materials S1.pdf]

Supplementary Materials S1

Delphi study Survey – Round 1

Section 1:

This section of questions contains a list of workplace risk factors that are associated with burnout in veterinary nurses and technicians. For each statement, you will be asked to indicate the ease with which you believe the risk factor can be addressed and then respond to three questions seeking your opinion on how the risk factor can be most effectively managed.

The following questions will be linked with each risk factor in the questionnaire:

Please indicate how easy, or difficult, you believe it is to address this issue:

1) very difficult
2) difficult
3) neither difficult nor easy
4) easy
5) very easy

Please answer the following questions, providing as much detail as you can:

1) What, if any, barriers are you aware of that make it difficult for veterinary nurse and technician managers or leaders to address this risk factor? [text box]
2) What, if any, enabling factors are you aware of that help to support veterinary nurse and technician managers or leaders to address this issue? [text box]
3) What solutions or strategies are you aware of that managers or leaders can implement to effectively address this issue? [text box]

Risk factors:

1) The workload is too high.
2) There is a lack of opportunities to utilise skills and knowledge for which veterinary nurses/technicians are trained and qualified.
3) A negative team culture exists (for example: bullying, gossiping, criticism, or general incivility).
4) There is a lack of, or unclear communication from both management and within the team.
5) There is poor management/leadership of the team (for example: micromanagement, favouritism, lack of support, or lack of action on team conflict).
6) There is an expectation of working overtime, not having a break, and a general lack of flexibility in rostering.
7) Remuneration is poor.

8) There is a lack of opportunity for progression or development.

9) Having to deal with rude or abusive clients.

10) There is a lack of appreciation, feeling valued, or being heard, by management.

Section 2:

This section of questions contains a list of statements around workplace factors that have been found to be protective for burnout in veterinary nurses and technicians, i.e. workplace factors that can help individuals to better cope with the stress associated with burnout. For each statement, you will be asked to indicate the ease with which you believe the protective factor can be promoted, or leveraged, within the workplace and then respond to three questions seeking your opinion on ways in which each factor can be most effectively promoted or utilised by managers or leaders to support their teams.

The following questions will be linked with each protective factor in the questionnaire:

Please indicate how easy, or difficult, you believe it is to promote this factor:

1) very difficult
2) difficult
3) neither difficult nor easy
4) easy
5) very easy

Please answer the following question, providing as much detail as you can:

1) What, if any, barriers are you aware of that make it difficult for veterinary nurse and technician managers or leaders to promote this factor? [text box]

2) What, if any, enabling factors are you aware of that help to support veterinary nurse and technician managers or leaders to promote this factor? [text box]

3) What strategies are you aware of that managers or leaders can implement to effectively promote this factor? [text box]

Protective factors:

1) Having some control over the schedule or expected tasks

2) Knowledge of having a positive impact on a patient or client

3) Being trusted with, and involved in, decisions around patient care
