# Supplementary material for: Workplace Strategies to Reduce Burnout in Veterinary Nurses and Technicians: A Delphi Study
Source: Animals (Basel). 2025 Apr 29;15(9):1257. doi: 10.3390/ani15091257 (PMC12071012; doi:10.3390/ani15091257)
Supplement: Supplementary file 1 [file animals-15-01257-s001.zip › Supplementary materials S2.pdf]

**Supplementary Materials S2**

**Delphi study Survey – Round 2**

**Section 1:**

This section includes solution statements that have been developed based on the panels' responses to the questions asked in round 1. For each risk factor you will be asked to indicate the ease with which you believe the proposed solution/s can be implemented, and how effective you believe the solution/s will be in addressing the risk factor. After considering the proposed solutions we would also like you to provide any further information or suggestions on how these solutions may be improved or any additional solutions you believe may be worth considering to address the risk factor.

For each solution statement, examples of actions have been included to provide further clarity, where required, on how this solution may be approached. These may be more applicable in different settings (e.g. corporate vs small private clinic).

Please answer the following questions, and provide as much detail as you can:

| **Risk factor 1: The workload is too high** | | | |
|---|---|---|---|
| **Proposed solutions** | **Please rate how easy or difficult you believe it will be to implement this solution** | **Please rate how effective or ineffective you believe this solution will be in addressing the risk factor** | **Examples of actions that may be implemented include:** |
| Hire more staff | Very Difficult 1 ○ 2 ○ 3 ○ 4 ○ 5 ○ Very Easy | Very Ineffective 1 ○ 2 ○ 3 ○ 4 ○ 5 ○ Very Effective | *Hire more VNTs and/or support staff to reduce non-clinical workload* |
| Improve staff retention | Very Difficult 1 ○ 2 ○ 3 ○ 4 ○ 5 ○ Very Easy | Very Ineffective 1 ○ 2 ○ 3 ○ 4 ○ 5 ○ Very Effective | *Improve workplace wellbeing, culture, leadership, staff training and utilisation* |
| Implement workload management systems to enhance efficiency and | Very Difficult 1 ○ 2 ○ 3 ○ 4 ○ 5 ○ Very Easy | Very Ineffective 1 ○ 2 ○ 3 ○ 4 ○ 5 ○ Very Effective | *Clear policies and SOPs to provide guidance on caseload and booking protocols; regular team meetings* |

| | | | |
|---|---|---|---|
| communications around workload issues | | | |
| Preventative healthcare focus to reduce the number of cases seen | Very Difficult 1 ○ 2 ○ 3 ○ 4 ○ 5 ○ Very Easy | Very Ineffective 1 ○ 2 ○ 3 ○ 4 ○ 5 ○ Very Effective | *Public and client education campaigns* |
| Please provide details of any modifications you believe would make these solutions easier to implement or more effective, or any additional solutions you believe may be worth considering to address this risk factor [text box] | | | |

| **Risk factor 2: There is a lack of opportunities to utilise skills and knowledge for which veterinary nurses/technicians are trained and qualified** | | | |
|---|---|---|---|
| **Proposed solutions** | **Please rate how easy or difficult you believe it will be to implement this solution** | **Please rate how effective or ineffective you believe this solution will be in addressing the risk factor** | **Examples of actions that may be implemented include:** |
| Provide role clarity on skill level and task expectations of VNTs and veterinarians | Very Difficult 1 ○ 2 ○ 3 ○ 4 ○ 5 ○ Very Easy | Very Ineffective 1 ○ 2 ○ 3 ○ 4 ○ 5 ○ Very Effective | *Clear SOPs outlining veterinary and VNT tasks and expectations; VNT levelling system based on education and experience* |
| Support veterinarians to delegate tasks to VNTs | Very Difficult 1 ○ 2 ○ 3 ○ 4 ○ 5 ○ Very Easy | Very Ineffective 1 ○ 2 ○ 3 ○ 4 ○ 5 ○ Very Effective | *Leadership support; veterinary training on VNT education and relevant legislation/regulations; delegation training* |
| Create opportunities for skill utilisation by reducing non-clinical workload and increasing clinical work opportunities | Very Difficult 1 ○ 2 ○ 3 ○ 4 ○ 5 ○ Very Easy | Very Ineffective 1 ○ 2 ○ 3 ○ 4 ○ 5 ○ Very Effective | *Introduce VNT clinics; technology to manage non-clinical workload; progression ladders for VNTs* |
| Implement systems to support delegation | Very Difficult 1 ○ 2 ○ 3 ○ 4 ○ 5 ○ Very Easy | Very Ineffective 1 ○ 2 ○ 3 ○ 4 ○ 5 ○ Very Effective | *VNT-to-patient and vet ratios; mentoring programs; clear SOPs for clinical tasks; support staff for non-clinical tasks; team rounds and collaboration* |
| Please provide details of any modifications you believe would make these solutions easier to implement or more effective, or any additional solutions you believe may be worth considering to address this risk factor [text box] | | | |

| Risk factor 3: A negative team culture exists (for example: bullying, gossiping, criticism, or general incivility) | | | |
|---|---|---|---|
| **Proposed solutions** | **Please rate how easy or difficult you believe it will be to implement this solution** | **Please rate how effective or ineffective you believe this solution will be in addressing the risk factor** | **Examples of actions that may be implemented include:** |
| Zero-tolerance to incivility at all levels of the workplace | Very Difficult 1 ○ 2 ○ 3 ○ 4 ○ 5 ○ Very Easy | Very Ineffective 1 ○ 2 ○ 3 ○ 4 ○ 5 ○ Very Effective | *Address issues early; support calling out unacceptable behaviour; disciplinary procedures and termination if necessary* |
| Promote culture change | Very Difficult 1 ○ 2 ○ 3 ○ 4 ○ 5 ○ Very Easy | Very Ineffective 1 ○ 2 ○ 3 ○ 4 ○ 5 ○ Very Effective | *Develop team vision and values collaboratively; culture officer role; psychological safety; regular and transparent communication; leadership support* |
| Set clear expectations on expected behaviour | Very Difficult 1 ○ 2 ○ 3 ○ 4 ○ 5 ○ Very Easy | Very Ineffective 1 ○ 2 ○ 3 ○ 4 ○ 5 ○ Very Effective | *Clear policies and definitions of bullying; codes of conduct; performance improvement plans; disciplinary procedures* |
| Provide staff and leadership training and support | Very Difficult 1 ○ 2 ○ 3 ○ 4 ○ 5 ○ Very Easy | Very Ineffective 1 ○ 2 ○ 3 ○ 4 ○ 5 ○ Very Effective | *On areas such as communication skills; conflict management; mental health first aid; civility training; human resources management; Diversity, Equity and Inclusion (DEI) awareness* |
| Please provide details of any modifications you believe would make these solutions easier to implement or more effective, or any additional solutions you believe may be worth considering to address this risk factor [text box] | | | |

| Risk factor 4: There is a lack of, or unclear, communication from both management and within the team | | | |
|---|---|---|---|
| **Proposed solutions** | **Please rate how easy or difficult you believe it will be to implement this solution** | **Please rate how effective or ineffective you believe this solution will be in addressing the risk factor** | **Examples of actions that may be implemented include:** |
| Increase communication opportunities | Very Difficult 1 ○ 2 ○ 3 ○ 4 ○ 5 ○ Very Easy | Very Ineffective 1 ○ 2 ○ 3 ○ 4 ○ 5 ○ Very Effective | *Inter-disciplinary and individual team meetings; team huddles and debriefs at the start and end of the day; leadership open door policy; feedback opportunities* |
| Promote and reward good communication | Very Difficult 1 ○ 2 ○ 3 ○ 4 ○ 5 ○ Very Easy | Very Ineffective 1 ○ 2 ○ 3 ○ 4 ○ 5 ○ Very Effective | *Leadership modelling; communication training; celebrate effective communication; psychological safety* |
| Develop clear communication protocols and reporting lines | Very Difficult 1 ○ 2 ○ 3 ○ 4 ○ 5 ○ Very Easy | Very Ineffective 1 ○ 2 ○ 3 ○ 4 ○ 5 ○ Very Effective | *Clear reporting lines; single source of information; communication templates; availability of recordings or minutes for those unable to attend* |
| Utilise different communication methods | Very Difficult 1 ○ 2 ○ 3 ○ 4 ○ 5 ○ Very Easy | Very Ineffective 1 ○ 2 ○ 3 ○ 4 ○ 5 ○ Very Effective | *Anonymous and identified feedback systems; technology to increase accessibility; centralised communication hub; seek input on individual preferences for communication* |
| Please provide details of any modifications you believe would make these solutions easier to implement or more effective, or any additional solutions you believe may be worth considering to address this risk factor [text box] | | | |

| Risk factor 5: There is poor management/leadership of the team (for example: micromanagement, favouritism, lack of support, or lack of action on team conflict) | | | |
|---|---|---|---|
| **Proposed solutions** | **Please rate how easy or difficult you believe it will be to implement this solution** | **Please rate how effective or ineffective you believe this solution will be in addressing the risk factor** | **Examples of actions that may be implemented include:** |
| Improve leadership recruitment and training processes | Very Difficult 1 ○ 2 ○ 3 ○ 4 ○ 5 ○ Very Easy | Very Ineffective 1 ○ 2 ○ 3 ○ 4 ○ 5 ○ Very Effective | *Recruitment strategies focused on leadership skills not clinical seniority; succession planning; provide ongoing training in leadership and human resource management* |
| Implement systems to support leaders | Very Difficult 1 ○ 2 ○ 3 ○ 4 ○ 5 ○ Very Easy | Very Ineffective 1 ○ 2 ○ 3 ○ 4 ○ 5 ○ Very Effective | *Rostered admin time; empowerment and support from upper management; role modelling and mentorship programs* |
| Improve workplace communication | Very Difficult 1 ○ 2 ○ 3 ○ 4 ○ 5 ○ Very Easy | Very Ineffective 1 ○ 2 ○ 3 ○ 4 ○ 5 ○ Very Effective | *Psychological safety; feedback systems; regular team meetings; transparency* |
| Initiate leadership reviews and accountability | Very Difficult 1 ○ 2 ○ 3 ○ 4 ○ 5 ○ Very Easy | Very Ineffective 1 ○ 2 ○ 3 ○ 4 ○ 5 ○ Very Effective | *Clear expectations; 360 reviews; performance management* |
| Please provide details of any modifications you believe would make these solutions easier to implement or more effective, or any additional solutions you believe may be worth considering to address this risk factor [text box] | | | |

| Risk factor 6: There is an expectation of working overtime, not having a break, and a general lack of flexibility in rostering | | | |
|---|---|---|---|
| **Proposed solutions** | **Please rate how easy or difficult you believe it will be to implement this solution** | **Please rate how effective or ineffective you believe this solution will be in addressing the risk factor** | **Examples of actions that may be implemented include:** |
| Review and adjust staffing to meet clinic needs | Very Difficult 1 ○ 2 ○ 3 ○ 4 ○ 5 ○ Very Easy | Very Ineffective 1 ○ 2 ○ 3 ○ 4 ○ 5 ○ Very Effective | *Hire more VNTs and/or support staff to meet workload; flexible or part time roles to increase coverage at busy periods; float VNTs to cover breaks* |
| Implement clear break and overtime policies | Very Difficult 1 ○ 2 ○ 3 ○ 4 ○ 5 ○ Very Easy | Very Ineffective 1 ○ 2 ○ 3 ○ 4 ○ 5 ○ Very Effective | *Rostered break times; on call schedule in the event overtime is required; casual VNT bank for last minute support* |
| Provide leadership and team training | Very Difficult 1 ○ 2 ○ 3 ○ 4 ○ 5 ○ Very Easy | Very Ineffective 1 ○ 2 ○ 3 ○ 4 ○ 5 ○ Very Effective | *Cross-training of team skills to enable greater support; communication and teamwork; wellness; workplace legislation; leadership training on turnover costs and effective workload management* |
| Review and implement workflow systems to streamline tasks and develop contingency plans | Very Difficult 1 ○ 2 ○ 3 ○ 4 ○ 5 ○ Very Easy | Very Ineffective 1 ○ 2 ○ 3 ○ 4 ○ 5 ○ Very Effective | *Limited service/bypass protocols (seeing only critical patients and redirecting non-critical cases elsewhere); team pipelines to improve workflow; clear policies to prevent overbooking; morning team huddles to plan workflow* |
| Please provide details of any modifications you believe would make these solutions easier to implement or more effective, or any additional solutions you believe may be worth considering to address this risk factor [text box] | | | |

| **Risk factor 7: Remuneration is poor** | | | |
|---|---|---|---|
| **Proposed solutions** | **Please rate how easy or difficult you believe it will be to implement this solution** | **Please rate how effective or ineffective you believe this solution will be in addressing the risk factor** | **Examples of actions that may be implemented include:** |
| Offer non-monetary remuneration | Very Difficult 1 ○ 2 ○ 3 ○ 4 ○ 5 ○ Very Easy | Very Ineffective 1 ○ 2 ○ 3 ○ 4 ○ 5 ○ Very Effective | *In consultation with individual staff members as suited to their needs (e.g. childcare support; travel vouchers; time off for study)* |
| Explore opportunities to increase revenue | Very Difficult 1 ○ 2 ○ 3 ○ 4 ○ 5 ○ Very Easy | Very Ineffective 1 ○ 2 ○ 3 ○ 4 ○ 5 ○ Very Effective | *Introduce new services (e.g. VNT clinics, telehealth); ensure correct charging; price increases; client education on pet insurance; budget forecasting* |
| Implement salary banding and progression pathways | Very Difficult 1 ○ 2 ○ 3 ○ 4 ○ 5 ○ Very Easy | Very Ineffective 1 ○ 2 ○ 3 ○ 4 ○ 5 ○ Very Effective | *Clear job descriptions and skill levels; pay commensurate with level; progression pathways and support to increase responsibility and skill level* |
| Implement work processes to reduce costs | Very Difficult 1 ○ 2 ○ 3 ○ 4 ○ 5 ○ Very Easy | Very Ineffective 1 ○ 2 ○ 3 ○ 4 ○ 5 ○ Very Effective | *Technology to automate routine tasks; appropriate utilisation of VNTs to increase veterinarian capacity;* |
| Please provide details of any modifications you believe would make these solutions easier to implement or more effective, or any additional solutions you believe may be worth considering to address this risk factor [text box] | | | |

| **Risk factor 8: There is a lack of opportunity for progression or development** | | | |
|---|---|---|---|
| **Proposed solutions** | **Please rate how easy or difficult you believe it will be to implement this solution** | **Please rate how effective or ineffective you believe this solution will be in addressing the risk factor** | **Examples of actions that may be implemented include:** |
| Develop clear progression pathways for VNTs | Very Difficult 1 ○ 2 ○ 3 ○ 4 ○ 5 ○ Very Easy | Very Ineffective 1 ○ 2 ○ 3 ○ 4 ○ 5 ○ Very Effective | *Clear progression bands; growth ladders; non-leadership progression pathways; clear VNT job descriptions with training to support growth within the clinic* |
| Explore professional growth opportunities | Very Difficult 1 ○ 2 ○ 3 ○ 4 ○ 5 ○ Very Easy | Very Ineffective 1 ○ 2 ○ 3 ○ 4 ○ 5 ○ Very Effective | *Areas for increased responsibility; undertaking specialised qualifications that can be used by the clinic; specific roles that match individuals' interests or skills* |
| Provide internal VNT training and support | Very Difficult 1 ○ 2 ○ 3 ○ 4 ○ 5 ○ Very Easy | Very Ineffective 1 ○ 2 ○ 3 ○ 4 ○ 5 ○ Very Effective | *Leadership; mentoring/coaching programs; individual development plans; implement a development and training officer role; support time off for study* |
| Promote external VNT training and support | Very Difficult 1 ○ 2 ○ 3 ○ 4 ○ 5 ○ Very Easy | Very Ineffective 1 ○ 2 ○ 3 ○ 4 ○ 5 ○ Very Effective | *Identify networking opportunities; signpost funding opportunities and access to CE; approach other industry members for mentoring (e.g. VTS)* |
| Please provide details of any modifications you believe would make these solutions easier to implement or more effective, or any additional solutions you believe may be worth considering to address this risk factor [text box] | | | |

| **Risk factor 9: Having to deal with rude or abusive clients** | | | |
|---|---|---|---|
| **Proposed solutions** | **Please rate how easy or difficult you believe it will be to implement this solution** | **Please rate how effective or ineffective you believe this solution will be in addressing the risk factor** | **Examples of actions that may be implemented include:** |
| Provide clear expectations on client conflict management and empower the team | Very 1 2 3 4 5 Very<br>Difficult ○ ○ ○ ○ ○ Easy | Very 1 2 3 4 5 Very<br>Ineffective ○ ○ ○ ○ ○ Effective | *Zero tolerance for abuse of staff; clear guidelines on what constitutes acceptable and non-acceptable behaviour; guidelines on when to escalate issues; communication protocols for conflict situations* |
| Create workplace support systems for VNTs faced with client abuse | Very 1 2 3 4 5 Very<br>Difficult ○ ○ ○ ○ ○ Easy | Very 1 2 3 4 5 Very<br>Ineffective ○ ○ ○ ○ ○ Effective | *Workplace culture; leadership support; employ social workers in the team; debriefs after difficult interactions; reporting systems for incidents* |
| Prepare and train the team for conflict situations | Very 1 2 3 4 5 Very<br>Difficult ○ ○ ○ ○ ○ Easy | Very 1 2 3 4 5 Very<br>Ineffective ○ ○ ○ ○ ○ Effective | *Never rostered alone; security systems in place (e.g. CCTV, duress alarms); provide regular training in de-escalation; develop client information on external financial and emotional support services* |
| Communicate clear behavioural expectations to clients | Very 1 2 3 4 5 Very<br>Difficult ○ ○ ○ ○ ○ Easy | Very 1 2 3 4 5 Very<br>Ineffective ○ ○ ○ ○ ○ Effective | *Code of conduct contracts; waiting room signage; transparency on costs and wait times; outline the consequences of abusing staff* |
| Please provide details of any modifications you believe would make these solutions easier to implement or more effective, or any additional solutions you believe may be worth considering to address this risk factor [text box] | | | |

| **Risk factor 10: There is a lack of appreciation, feeling valued, or being heard, by management** | | | |
|---|---|---|---|
| **Proposed solutions** | **Please rate how easy or difficult you believe it will be to implement this solution** | **Please rate how effective or ineffective you believe this solution will be in addressing the risk factor** | **Examples of actions that may be implemented include:** |
| Implement VNT recognition systems | Very Difficult 1 ○ 2 ○ 3 ○ 4 ○ 5 ○ Very Easy | Very Ineffective 1 ○ 2 ○ 3 ○ 4 ○ 5 ○ Very Effective | *Newsletters; VNT of the month; long service awards; staff appreciation days; staff appreciation committee; standing agenda item in meetings* |
| Increase communication channels between management and VNTs | Very Difficult 1 ○ 2 ○ 3 ○ 4 ○ 5 ○ Very Easy | Very Ineffective 1 ○ 2 ○ 3 ○ 4 ○ 5 ○ Very Effective | *Feedback systems; regular meetings; utilise multiple communication channels; open door policies; Town Halls* |
| Provide support and training for leaders | Very Difficult 1 ○ 2 ○ 3 ○ 4 ○ 5 ○ Very Easy | Very Ineffective 1 ○ 2 ○ 3 ○ 4 ○ 5 ○ Very Effective | *On areas such as effective communication; emotional intelligence; Diversity, Equity and Inclusion (DEI); culture; develop mentoring and peer group programs* |
| Identify what appreciation looks like for individuals | Very Difficult 1 ○ 2 ○ 3 ○ 4 ○ 5 ○ Very Easy | Very Ineffective 1 ○ 2 ○ 3 ○ 4 ○ 5 ○ Very Effective | *Survey the team; individual development plans; provide dedicated time for leaders to spend with staff* |
| Please provide details of any modifications you believe would make these solutions easier to implement or more effective, or any additional solutions you believe may be worth considering to address this risk factor [text box] | | | |

**Section 2:**

This section includes protective factor promotion strategies that have been developed based on the panels' responses to the questions asked in round 1. For each protective factor promotion strategy statement, you will be asked to indicate the ease with which the strategy can be implemented. After considering the proposed strategies, we would also like you to provide any further information or suggestions on how these strategies may be improved or any additional strategies you believe may be worth considering to leverage the mitigating impact this factor has on burnout.

For each promotion strategy statement, examples of actions have been included to provide further clarity, where required, on how this strategy may be approached. These may be more applicable in different settings (e.g. corporate vs small private clinic).

| **Protective Factor 1: Having some control over the schedule or expected tasks** | | |
|---|---|---|
| **Proposed strategies** | **Please rate how easy or difficult you believe it will be to implement this strategy** | **Examples of actions that may be implemented include:** |
| Adopt a collaborative team scheduling approach | Very Difficult 1 ○ 2 ○ 3 ○ 4 ○ 5 ○ Very Easy | *VNT representation in management decision making; morning team huddles to plan tasks, breaks, and teamwork; support innovation to improve scheduling or workflow practices* |
| Upskill and cross-train VNTs | Very Difficult 1 ○ 2 ○ 3 ○ 4 ○ 5 ○ Very Easy | *Increase capacity to perform in all VNT roles; build confidence to work autonomously; cross-train to increase opportunity to swap shifts;* |
| Develop clear expectations for VNT tasks and roles | Very Difficult 1 ○ 2 ○ 3 ○ 4 ○ 5 ○ Very Easy | *Collaborative development of VNT shift descriptions with tasks and skill levels outlined* |
| Implement work systems to enhance clarity and communications around scheduling | Very Difficult 1 ○ 2 ○ 3 ○ 4 ○ 5 ○ Very Easy | *Clear booking policies to prevent overbooking; break and overtime policies; seek VNT feedback on scheduling issues and collaboratively problem solve* |
| Please provide details of any modifications you believe would make this strategy easier to implement or more effective, or any additional strategies you believe may be worth considering to promote this burnout protective factor [text box] | | |

| Protective Factor 2: Knowledge of having a positive impact on a patient or client | | |
|---|---|---|
| **Proposed strategies** | **Please rate how easy or difficult you believe it will be to implement this strategy** | **Examples of actions that may be implemented include:** |
| Implement peer feedback systems | Very Difficult 1 ○ 2 ○ 3 ○ 4 ○ 5 ○ Very Easy | *Kudos boards; team meeting shout outs; communication systems to pass feedback up to management; make acknowledgement part of team huddles and debriefs* |
| Encourage clear and open communication within the team | Very Difficult 1 ○ 2 ○ 3 ○ 4 ○ 5 ○ Very Easy | *Culture; leadership modelling of good communication; create regular and easy feedback systems and habits* |
| Provide recognition based on individual VNT needs | Very Difficult 1 ○ 2 ○ 3 ○ 4 ○ 5 ○ Very Easy | *Survey the team to determine preferences; public vs private feedback; generation and career stage influences* |
| Please provide details of any modifications you believe would make this strategy easier to implement or more effective, or any additional strategies you believe may be worth considering to promote this burnout protective factor [text box] | | |

| **Protective Factor 3: Being trusted with, and involved in, decisions around patient care** | | |
|---|---|---|
| **Proposed strategies** | **Please rate how easy or difficult you believe it will be to implement this strategy** | **Examples of actions that may be implemented include:** |
| Increase veterinarian and leadership awareness of VNT training and capabilities | Very Difficult 1 ○ 2 ○ 3 ○ 4 ○ 5 ○ Very Easy | *Encourage active involvement of VNTs in case discussions providing an opportunity to demonstrate knowledge; education of veterinarians and leaders on VNT education levels and professional regulation* |
| Develop clear VNT capability and advancement levels | Very Difficult 1 ○ 2 ○ 3 ○ 4 ○ 5 ○ Very Easy | *Clear guidelines around skills and abilities expected at each level; conduct VNT competency assessments to progress to next level; develop a spectrum of care chart to clearly outline clinical expectations at each level* |
| Build veterinarian trust in VNTs through fostering a collaborative culture | Very Difficult 1 ○ 2 ○ 3 ○ 4 ○ 5 ○ Very Easy | *Psychological safety; all team patient rounds; veterinarian to VNT mentoring and reverse mentoring programs; encourage questions and discussion for learning; team debriefing; leadership support; team consulting; collaborative patient care plans* |
| Support VNT professional development and learning | Very Difficult 1 ○ 2 ○ 3 ○ 4 ○ 5 ○ Very Easy | *Individual development plans; in house training; journal clubs; shadowing shifts with experienced VNTs; provide clear patient parameter ranges and alert guidelines to inexperienced VNTs* |
| Please provide details of any modifications you believe would make this strategy easier to implement or more effective, or any additional strategies you believe may be worth considering to promote this burnout protective factor [text box] | | |
